# Pharmacophore Virtual Screening Identifies Riboflavin as an Inhibitor of the Schistosome Cathepsin B1 Protease with Antiparasitic Activity

**DOI:** 10.1021/acsomega.4c03376

**Published:** 2024-05-30

**Authors:** Ramon M. Cogo, Thaís F. A. Pavani, Ana C. A. Mengarda, Rayssa A. Cajas, Thainá R. Teixeira, Lucas Fukui-Silva, Yujie Uli Sun, Lawrence J. Liu, Dilini K. Amarasinghe, Michael C. Yoon, Osvaldo A. Santos-Filho, Josué de Moraes, Conor R. Caffrey, Daniela G. G. Rando

**Affiliations:** † Universidade Federal de São Paulo—Campus Diadema, Curso de Pós-Graduação em Biologia Química da Unifesp, Rua São Nicolau 210, 2o andar, Centro, Diadema, São Paulo 09972-270, Brazil; ‡ Universidade Guarulhos, Núcleo de Pesquisa em Doenças Negligenciadas—NPDN, Praça Tereza Cristina 88, Guarulhos 09972-270, Brazil; § Center for Discovery and Innovation in Parasitic Diseases, Skaggs School of Pharmacy and Pharmaceutical Sciences, University of California San Diego, La Jolla, California 92093-0021, United States; ∥ Instituto de Pesquisas de Produtos Naturais Walter Mors, Universidade Federal do Rio de Janeiro, Av. Carlos Chagas Filho, 373, Bloco H, Rio de Janeiro 21941-853, Brazil; ⊥ Grupo de Pesquisas Químico-Farmacêuticas da Unifesp, Department of Pharmaceutical Sciences Rua São Nicolau, Universidade Federal de São Paulo—Campus Diadema, 210, 2o andar, Centro, Diadema, São Paulo 09972-270, Brazil

## Abstract

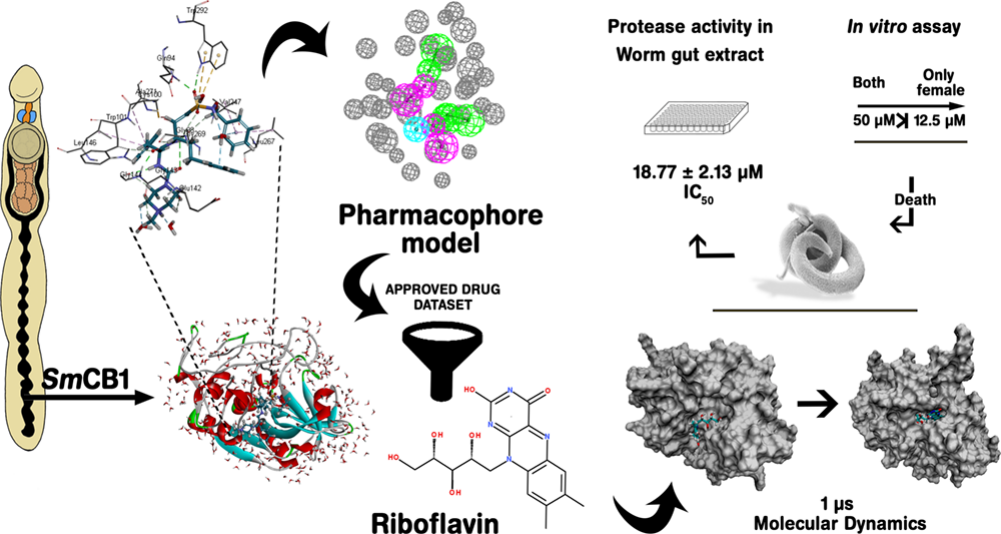

Schistosomiasis is a neglected disease of poverty that affects over 200 million people worldwide and relies on a single drug for therapy. The cathepsin B1 cysteine protease (SmCB1) of *Schistosoma mansoni* has been investigated as a potential target. Here, a structure-based pharmacophore virtual screening (VS) approach was used on a data set of approved drugs to identify potential antischistosomal agents targeting SmCB1. Pharmacophore (PHP) models underwent validation through receiver operating characteristics curves achieving values >0.8. The data highlighted riboflavin (RBF) as a compound of particular interest. A 1 μs molecular dynamics simulation demonstrated that RBF altered the conformation of SmCB1, causing the protease’s binding site to close around RBF while maintaining the protease’s overall integrity. RBF inhibited the activity of SmCB1 at low micromolar values and killed the parasite in vitro. Finally, in a murine model of *S. mansoni* infection, oral administration of 100 mg/kg RBF for 7 days significantly decreased worm burdens by ∼20% and had a major impact on intestinal and fecal egg burdens, which were decreased by ∼80%.

## 1 Introduction

Helmintic diseases caused by nematodes and flatworms affect almost 1.8 billion people worldwide, predominantly in regions characterized by insufficient health and social and economic conditions.^1−3^ Among flatworm species, the blood fluke of the genus *Schistosoma* alone is responsible for nearly 240 million cases globally, with 700 million people at risk of infection.^4^ Three species of schistosomes are medically important, *S. mansoni* and *S. japonicum*, which cause intestinal schistosomiasis, and *S. hematobium*, which is associated with various pathologies associated with the urogenital system.^5^

Praziquantel (PZQ) is the current World Health Organization-recommended treatment for schistosomiasis. After 40 years of mass drug administration, PZQ is safe and reasonably effective in decreasing disease-related morbidity.^6−8^ However, in the single dose offered, cure is rare, and the drug has pharmaceutical and pharmacological deficiencies. The dependence on a single drug to treat so prevalent a disease risks the emergence of resistance, especially when considering past experiences with other complex pathogens such as mycobacteria and some protozoa (malaria, leishmaniasis, Chagas disease).^9−13^

In response to this concern, researchers have explored various schistosome protein targets in the quest for new therapeutic alternatives.^14,15^ One of these is a cysteine protease known as cathepsin B1 (or SmCB1 in *S. mansoni*), which is primarily expressed in the worm’s digestive tract, where it contributes to the digestion of host blood proteins, including hemoglobin, as a source of nutrients.^16−18^ Inhibition of SmCB1 expression via RNA interference (RNAi) slowed the growth of the parasite in vitro, underscoring the enzyme’s importance to the parasite’s development.^19^ Further, in a mouse model of *S. mansoni* infection, a small molecule inhibitor of cysteine proteases, K11777, decreased worm and egg burdens, which was associated with the engagement by the inhibitor of SmCB1.^20^

Since then, SmCB1 has been the focus of reports involving its cocrystallography and quantum mechanics studies with various peptidyl inhibitors, principally of the vinyl sulfone^21−23^ and azanitrile reactive groups^24^ (warheads). These studies have provided crucial information on the binding modes of these ligands, which will aid in the discovery of new SmCB1 inhibitors with mechanisms of action that are distinct from PZQ,^25^ thus satisfying one of the tenets of the desired product profile for new schistosomiasis drugs.^26,27^

Accordingly, using the structural insights reported above for irreversible inhibitors, we designed and executed a pharmacophore-based virtual screening (VS) strategy to discover new SmCB1 inhibitors from a database of drugs approved for use in humans by the US Food and Drug Administration. By integrating VS with drug repositioning, we aimed to reduce both the time and costs associated with the drug discovery process, which is important when it comes to drugs for diseases associated with poverty.

## 2 Material and Methods

### 2.1 Protein and Ligand Preparation

The cocrystal structure of SmCB1 covalently bound to the WRR-286 (PDB ID: 5OGR; resolution 1.55 Å)^28^ was used as the basis for this study. Hydrogen atoms were added, taking into account a pH value of 5.5, in accordance with the enzyme inhibition assay performed (IC_50_ values).^22^ Energy minimization was carried out with a 0.1 RMS gradient threshold using the Steepest Descent algorithm.^29^ The covalent bond between the β-carbon of WRR-286’s vinyl sulfonic group and the sulfur of Cys100 was manually broken. Subsequently, the double bond between the α- and β-carbons of WRR-286’s vinyl sulfonic group was restored, followed by the addition of a hydrogen atom to the thiol group of Cys100.^23^

The GaussView 5.0.1^30^ software was used to model previously described inhibitors of SmCB1. The vinyl sulfone analogues were designed based on the scaffold structure of the cocrystallized WRR-286,^22^ whereas the thiosemicarbazones^31^ were redrawn. All structures were energy-minimized with Gaussian 09W software^32^ using the Hartree–Fock level of theory^33^ and the 6-31G* basis set.^34,35^

### 2.2 Pharmacophore Modeling

Using the Discovery Studio 2021 software,^36^ the intermolecular interactions between SmCB1 and WRR-286 were used for pharmacophore modeling. Initially, both the ligand and enzyme were reparametrized using the MMFF^37^ and CHARMPLR^38^ force fields, respectively. Water molecules participating in the intermolecular interactions network (water–water, water–enzyme, and WRR-286-water-enzyme hydrogen bonds) were kept during the pharmacophore modeling.^28^ Ten pharmacophore models were generated, each consisting of a minimum of four and a maximum of six features.^39^ The validation process involved the construction of receiver operating characteristics^40^ (ROC) (Table S1—Supporting Information) curves and the calculation of Areas under the Curves (AUC-ROC). Models with AUC-ROC values exceeding 0.7 were considered to be suitable. Sets of known ligands (active compounds and modeled decoys, along with nonactive ligands) were used as references to challenge the models. A conformational search method, namely “BEST,” implemented in Discovery Studio software and which utilizes the poling technique to induce conformational variation,^41−43^ generated a maximum of 100 flexible conformers with energy differences not exceeding 20 kcal/mol. Moreover, as a steric feature, the method “excluded volumes” was set to a maximum distance of 4.5 Å.

### 2.3 Data Sets

All data sets (Supporting Information—Figure S1) were prepared by removing duplicates and generating tautomers, isomers, and protonation states.

The validation data sets were constructed using cathepsin B inhibitors obtained from BindingDB,^44,45^ Jílkova et al.^22^ and Fonseca et al.,^31^ as well as from the decoys generated through the Database of Useful Decoys (DUD-E) web-based software.^46^ The BindingDB data set search was performed to find general and experimentally validated cathepsin B1 ligands with measured inhibitor constants (*K_i_*). Unsuitable compounds were manually removed based on the following criteria: (i) compounds reported in more than one literature source and for which there is a difference in *K_i_* values >10 nM for the same enzyme; (ii) compounds with activities measured for orthologous enzymes from different organisms (e.g., *Homo sapiens*, *Bos taurus*) and for which the *K_i_* values differ significantly from each other; (iii) compounds with equal DOI but different *K_i_* values. Compounds with *K_i_* values under 100 nM were selected to create the “true active” instances, along with the known active vinyl sulfone and thiosemicarbazone analogues described by Jílková et al.^22^ and Fonseca et al.,^31^ respectively.

DUD-E was used to model a set of decoys. To ensure physicochemical similarity, the 20 vinyl sulfone analogs^22^ were used as references for this modeling. Furthermore, to prevent analogue bias, the generated structures included large compounds that would cause steric clashes in the binding site and molecules that are considered synthetically “impossible” due to their structural complexity.

The assembled “inactive” data set consisted of compounds from BindingDB with *K_i_* values >10 μM, the decoy set and 18 experimentally negative compounds synthesized by Fonseca et al.^31^ To evaluate the pharmacophore models generated, the BIOVIA Discovery Studio platform^36^ was used to reparametrize all data sets under the MMFF94 force field.^37^

### 2.4 Screening Library

An FDA data set comprising 2100 compounds was obtained from the ZINC15 database^47^ and processed as described above for the other compound data sets. Partial charges were estimated using the MMFF94 force field, and an energy minimization calculation was conducted to ensure a total energy gradient of 0.001 kcal/mol among the conformers.

### 2.5 Molecular Dynamics and Free Energy Calculations

CHARMM-GUI^48^ was used for the initial preparation of the GROMACS input files. The molecular dynamics simulations (MDS) were performed with the CHARMM36 force field,^49^ implemented in the GROMACS 2021 software.^50^ Riboflavin (RBF) was parametrized using CHARMM General Force Field (CGenFF v. 2.5.1).^51^ To solvate the system, a cubic water box was constructed, and the TIP3P water model was used within a radius of 10 Å from the center of the system. The counterions, Na and Cl, were added to neutralize the system charge. Periodic boundary conditions were also applied.

Both systems, i.e., the complex and the free protein, underwent energy minimization using the steepest descent algorithm for 50,000 steps, with a convergence criterion setup at 0.01 kcal/mol. Subsequently, the system was equilibrated over 5 ns, utilizing the NVT ensemble at a temperature of 303.15 K. Finally, the production phase was performed over 1 μs and employed the NPT ensemble with the Nosé–Hoover thermostat,^52^ and a constant temperature of 303.15 K. To maintain a constant pressure of 1 bar, the Parrinello–Rahman method^53^ was applied. Long-range interactions were calculated using the particle mesh Ewald^54^ method. Frames from the simulation were sampled every 100 ps.

The molecular mechanics/Poisson–Boltzmann surface area (MM/PBSA) method^55^ was used to calculate the free energy of binding. These calculations were performed using the gmx_mmpbsa tool.^56^ The binding energy can be decomposed into its individual components based on the residues (amino acids) of the macromolecular target. Initially, the *E*_MM_, *G*_polar_, and *G*_nonpolar_ energy components of each atom within each residue are computed for both the bound and unbound forms. Subsequently, the contribution to the interaction energy Δ*R*_*x*_^BE^ of the residue *x* is calculated as follows:
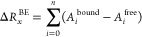
where *A*_*i*_^bound^ and *A*_*i*_^free^ represent the energy of atom *i* of residue *x* in the bonded and unbonded states, respectively, and *n* is the total number of atoms in the residue. The energy contribution from all residues sums up to the interaction energy, denoted as Δ*G*_binding_ = ∑_*x* = 0_^*m*^Δ*R*_*x*_^BE^, where *m* is the total number of residues comprising the ligand-protein complex.^57^

### 2.6 Inhibition of SmCB1 and Human Cathepsin B by RBF

The SmCB1 assay is based on an established procedure using soluble extracts of adult *S. mansoni* (10 males and 10 females) prepared in 0.1 M sodium citrate, pH 5.0.^58,59^ Recombinant human cathepsin B (R&D Systems #953-CY) was activated by incubation in 25 mM MES, 5 mM DTT, pH 5.0, for 15 min at room temperature.

Proteolytic activity was measured at room temperature by hydrolysis of the peptidyl fluorogenic substrate, Z-Arg-Arg-7-amido-4-methylcoumarin (Z-RR-AMC; R&D Systems #ES008). In a 384-well black plate (Greiner #781091), enzyme (6.4 mg/mL *S. mansoni* extract or 1.33 μg/mL human cathepsin B) was preincubated with inhibitor for 15 min at room temperature in 30 μL 0.05 M sodium citrate buffer, pH 5.0, 2 mM DTT with or without 0.1% Triton X-100 (Sigma #T8787). An equal volume of the same buffer, containing 20 μM Z-RR-AMC, was added, and the relative fluorescence units (RFUs) were recorded at excitation and emission wavelengths of 360 and 460 nm, respectively, over 30 min in a BioTek Synergy HTX multimode reader. Enzyme velocity (RFU/sec) was calculated using the highest slope recorded for 10 consecutive readings and normalized by comparison to the rates of reaction of the DMSO control. IC_50_ curves were obtained by nonlinear regression using GraphPad Prism. Assays were performed twice each in triplicate.

A 12-point concentration range of riboflavin (3000–0.017 μM) was set up to measure its potential inhibition of SmCB1 and human cathepsin B. For the assay using worm extracts, a number of protease-class-specific inhibitors were tested as controls, specifically, the cysteine protease inhibitors, E-64 and leupeptin (each 10 μM final), and the serine protease inhibitor, AEBSF (2 mM final). The cathepsin B-specific inhibitor CA-074 (10 μM final) was also included as a control.

### 2.7 Antischistosomal Activity of RBF In Vitro and in an Animal Model of Infection

#### 2.7.1 Animals and Parasite Maintenance

The animal studies described here comply with the National Centre for the Replacement, Refinement, and Reduction of Animals in Research (NC3Rs) ARRIVE guidelines. The experimental design received approval from the Committee for the Ethical Use of Animals in Experimentation at Guarulhos University (Guarulhos, SP, Brazil; protocol ID 47/20) following the Guidelines for Care and Use of Laboratory Animals as stipulated by Brazilian laws.

*S. mansoni* (Belo Horizonte, BH strain) was maintained by passaging in Swiss mice and *Biomphalaria glabrata* snails at the Research Center on Neglected Diseases (Guarulhos, SP, Brazil).^60^ Both snails and mice were housed under controlled environmental conditions (25 °C, 50% humidity) and a 12 h light-dark cycle with free access to water and food. Infected snails were induced by light to release infectious larvae (cercariae), and mice were subcutaneously infected with 120 *S. mansoni* cercariae each. Rodents were randomly housed in individually vented caging systems, with five animals per cage.^61^

#### 2.7.2 In Vitro Antischistosomal Activity of RBF

Antischistosomal tests follow established methodologies.^62,63^ Seven-week-old adult *S. mansoni* were collected from the hepatic portal and mesenteric veins. The parasites were washed and then maintained in RPMI 1640 medium containing 10% fetal bovine serum, 100 IU·ml^–1^ penicillin, and 100 μg·ml^–1^ streptomycin, at 37 °C and 5% CO_2_. RBF, previously solubilized in DMSO, was tested at concentrations ranging from 50 to 6.25 μM in triplicate wells within a 24-well culture plate. DMSO at 0.5% (the highest concentration of the solvent) served as the negative control, while PZQ at 2 μM served as the positive control. After 24, 48, and 72 h, parasite viability was assessed using an inverted microscope.^64^

#### 2.7.3 Efficacy in a Murine Model of *S. mansoni* Infection

In vivo studies employed *S. mansoni*-infected mice following standard protocols.^63,65^ Three-week-old mice (Swiss) were subcutaneously infected with 80 *S. mansoni* cercariae. Compounds were administered 42 days postinfection using a single, 400 mg/kg oral dose or once daily 100 mg/kg doses for seven consecutive days. A single oral dose of 400 mg/kg PZQ (400 mg/kg), dissolved in 2% ethanol, served as the positive control, whereas mice treated with vehicle alone served as the negative control. Therapeutic efficacy was evaluated based on reductions in worm and egg burdens, the latter based on measuring fecal egg counts, and the oogram method^64^ to determine egg burden in the intestine. Statistical analyses employed GraphPad Prism (ver. 8.0; CA, USA). The percentage of worm and egg reduction was calculated, and the Kruskal–Wallis test compared medians between the treatment and control groups.^66^ Statistical significance was considered at *P* < 0.05.

## 3 Results and Discussion

### 3.1 SmCB1-WRR-286 Complex

VS was performed using data based on the binding parameters of a previously described set of vinyl sulfone derivatives.^22^ These compounds were designed to target cysteine proteases and their structures are similar to the peptidomimetic inhibitor Ac-NMrAF^21^ (Supporting Information—Figure S3), which mimics part of an α-helix region of the pro-peptide that inhibits the SmCB1 zymogen structure.^21,22^ This α-helix region is unique to SmCB1 and is not observed in the structurally related SmCB2 protease.^21^ Also, to further improve selectivity to SmCB1, the authors explored different groups that would interact with that protease’s S1 hydrophobic pocket (Supporting Information—Figure S3), due to the proximity of residues Trp292 and Gln94 from Ile193, which are present in a nonconserved segment of the occluding loop.^22^

The crystallographic complex between SmCB1 and WRR-286 (PDB code: 5OGR—1.55 A resolution) was employed to perform this study. A pretreatment of this complex was performed to check for any missing atoms or residues. It is important to highlight that the starting complex is in its covalent state. The proposed molecular process for covalently bound formation consists of ligand approximation of the recognition site, interaction through noncovalent forces (characterized by *K_i_*), and, finally, establishing a covalent σ bond which completely inactivates the enzyme.

Because the recognition process involves the noncovalent step, the bond between the β carbon of WRR-286 and the sulfur atom of Cys100 was manually broken. Then, the vinyl group between the α and β carbons of the ligand was restored, and the hydrogens were added to the Cys100 sulfur atom and the WRR-286 vinyl carbons. To relax the system, an energy minimization calculation was performed with a 0.1 kcal/mol/Å^2^ gradient. Finally, the ligand was then removed from the complex, reparametrized with the MMFF94 force field, and reinserted into the cavity.

### 3.2 Pharmacophore Modeling and Validation

Pharmacophore modeling returned 10 possible models for which the associated statistics were evaluated through the construction of ROC curves.^39^ Model validation is a crucial step in pharmacophore modeling and demands a careful approach with suitable data curation.^67–69^ Validation of the model employed two sets of compounds, the TA (total active compounds) and TI (total inactive compounds), to challenge the obtained model. This approach allows for the building a confusion matrix from which a ROC curve is plotted to analyze the model's capacity to recognize and discriminate between true positive and true negative compounds. From the ROC curve, the AUC-ROC can be calculated to evaluate the overall quality of the models.

The true positive (TP) library was constructed using established SmCB1 inhibitors, including 19 vinyl sulfones^22^ and seven thiosemicarbazones.^31^ Because these compounds alone were insufficient to build a robust TP library, additional CB inhibitors were sourced from the Binding Database. After VS, selectivity toward SmCB1 was assessed by considering interactions with residues or regions specific to the schistosome enzyme. Following the criteria outlined in Section 2, a final TP library comprising 188 compounds was used.

The true negative (TN) library was constructed using 101 compounds from the BindingDB data set, all of which had *K_i_* values >10 μM. These were combined with 18 compounds from the library studied by Fonseca et al.,^31^ which did not interact with SmCB1. In order to expand the library and ensure structural similarity with the vinyl sulfones, a set of 1609 decoys was generated using the web-based software, DUD-E, and incorporating 19 vinyl sulfones as references. The final TN library, therefore, consisted of 1482 compounds.

The imbalance between the two test libraries (188 vs. 1482 compounds) was chosen to simulate what occurs in nature. For both libraries, isomers, tautomers, and specific ionized forms, as well as 100 conformers of each compound, were considered during the validation approach. Pharmacophore modeling was then performed, and the 10 best models were retrieved. Table 1 lists the model statistical validation data as well as the estimated sensitivity, specificity, and AUC-ROC.

**Table 1:** Statistical Data Estimated for the Pharmacophore Models

model	total actives	total inactives	true positives	true negatives	false positives	false negatives	sensitivity	specificity	AUC-ROC
PHP_01	188	1482	159	922	560	29	0.84574	0.62213	0.823
PHP_02	188	1482	157	913	569	31	0.83511	0.61606	0.816
PHP_03	188	1482	150	1029	453	38	0.79787	0.69433	0.807
PHP_04	188	1482	153	1039	443	35	0.81383	0.70108	0.810
PHP_05	188	1482	154	1012	470	34	0.81915	0.68286	0.815
PHP_06	188	1482	153	930	552	35	0.81383	0.62753	0.798
PHP_07	188	1482	154	953	529	34	0.81915	0.64305	0.808
PHP_08	188	1482	158	928	554	30	0.84043	0.62618	0.816
PHP_09	188	1482	151	1038	444	37	0.80319	0.7004	0.809
PHP_10	188	1482	154	1045	437	34	0.81915	0.70513	0.812

**Figure 1:**
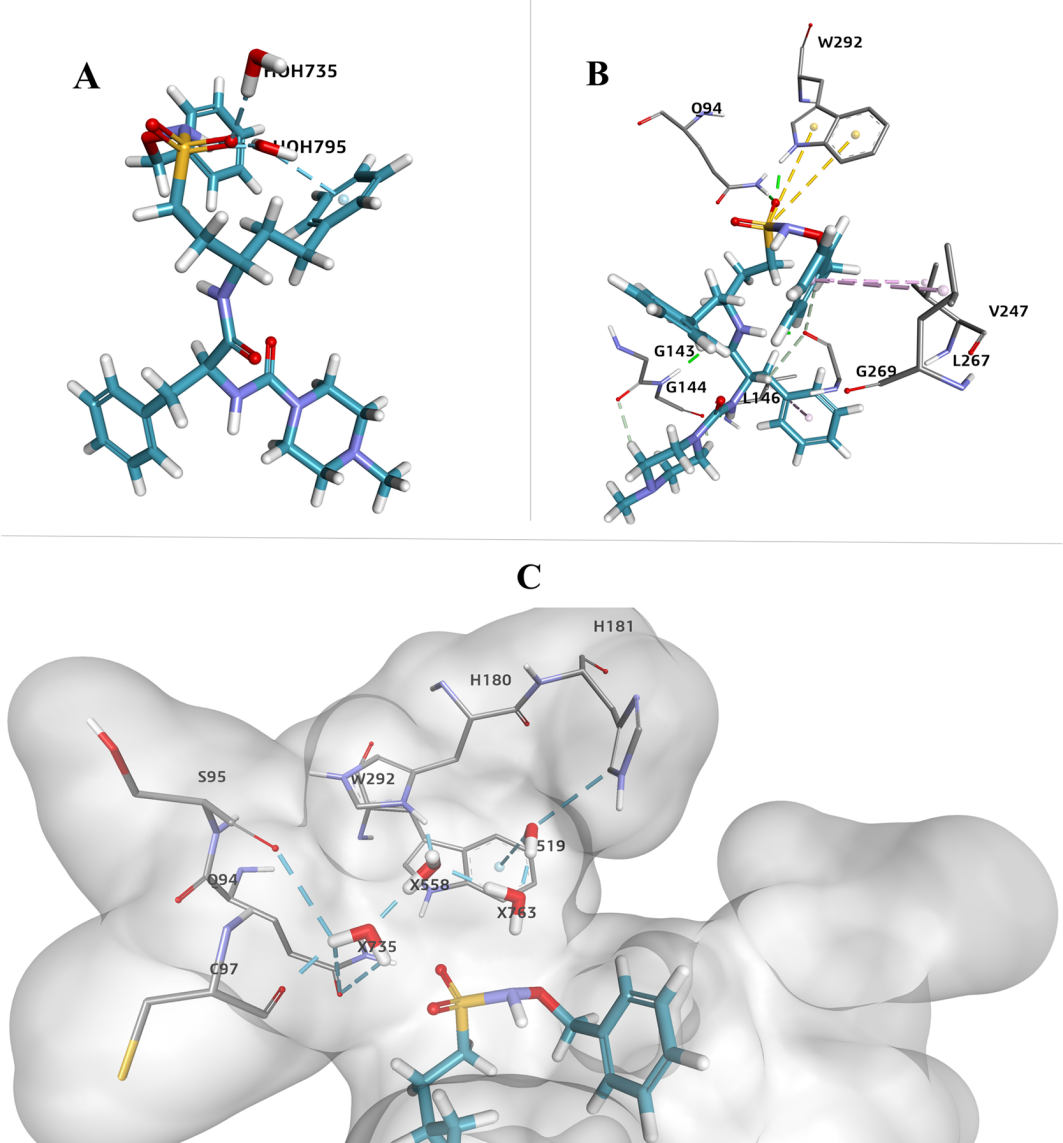
SmCB1 and WRR-286 interactions. (A) WRR-286 HOH; (B) residue interactions (90° rotation); and (C) water net interactions.

Of the ten models, nine presented AUC-ROC values higher than 0.8, the exception being PHP-06. All models, however, were able to correctly classify approximately 82 and 66% of the positive and negative compounds, respectively. The average discriminative power of the models to identify negative compounds did not achieve a high value, as can be seen in the specificity column of Table 1. Such a discriminative power is described in the false positives column, which represents a third of the total negatives (Table 1). Nevertheless, it is important to note that the main driving force in the recognition of WRR-286 by SmCB1 is the formation of a covalent bond with the target enzyme,^23^ an aspect that cannot be simulated through this pharmacophore modeling approach. Other modeling protocols, however, could be applied to find covalent inhibitors.^69,70^ Analysis of the interaction energies involved in the complex, however, revealed that the selected features employed to construct the models were derived from the lowest potential energy of the interaction between SmCB1 and WRR-286, namely, those established with Gly144, Gly269, Gly143, Gln94, Leu146, Leu267, Val247, Trp292, HOH735, and HOH 795 (Figure 1A,B; Table 2).

**Table 2:** Protein Residues-Related Pharmacophore Features

	features						
model	HBDon-02	HBDon-05	HBAc-29	HBAc-19	HBAc-20	HBDon-10	HYD
PHP-01	GLY144	GLY269	GLY143	HOH735	GLN94	HOH795	
PHP-02	GLY144	GLY269	GLY143	HOH735	TRP292	HOH795	
PHP-03	GLY144	GLY269		HOH735	GLN94	HOH795	LEU146
PHP-04	GLY144	GLY269		HOH735	GLN94	HOH795	LEU267VAL247
PHP-05	GLY144	GLY269		HOH735	GLN94	HOH795	HOH795
PHP-06	GLY144	GLY269	GLY143	HOH735		HOH795	LEU146
PHP-07	GLY144	GLY269	GLY143	HOH735		HOH795	LEU267VAL247
PHP-08	GLY144	GLY269	GLY143	HOH735		HOH795	HOH795
PHP-09	GLY144	GLY269		HOH735	TRP292	HOH795	LEU146
PHP-10	GLY144	GLY269		HOH735	TRP292	HOH795	LEU267VAL247

The interactions between ordered water molecules^71^ and the ligand appeared to be significant. The HOH735 and HOH795 water molecules interact directly with the ligand, but other molecules around them construct a water net that seems to stabilize the complex by establishing bridges between the ligand and other amino acid residues in the vicinity of the binding cavity (Figure 1C).

This phenomenon is aligned with the observations of Jílková et al.^28^ regarding a set of water molecules involved in an interaction network, which is present not only in this system but is also observed for other SmCB1 crystal structures, such as those obtained with WRR-391.^28^ For this reason, the water molecules were kept during the performance of the experiments. Additionally, the presence of waters in the binding site can enhance VS performance.^72^

The 10 models were also visually inspected by their biological significance, and five models were chosen to proceed with the VS since they presented the best sensitivity and specificity values, namely, PH-01, -05, -08, -09, and -10 (Supporting Information—Figure S2). All presented a set of common features, namely, HBDon-02, HBDon-05, HBAc-19, and HBDon-10, but differed in regard to the interactions between HBAc-14 and HBAc-20 and aromatic features (Supporting Information—Table S3).

### 3.3 Virtual Screening

VS was performed with a set of 255 conformers of each ligand from the FDA data set which were flexibly screened employing each of the five chosen pharmacophore models. Compounds that fulfilled four or more features per model were selected as potential ligands and retrieved as tridimensional coordinates associated with a fit value for further examination. The results were then filtered by considering the selection frequency of the compounds for each constructed model. Moreover, the presence of the same compound in more than one pharmacophore was evaluated by a consensus analysis.

Visual inspection of the ligand conformation retrieved from the pharmacophore model was performed to analyze its fit into the enzyme cavity and search for potential clashes. Cavity coordinates are preserved on the models, and since “pharmacophore fitness” does not always mean “cavity fitness”, it was important to perform this inspection. The pharmacophore feature “excluded volumes”^73^ (Supporting Information—Figure S2) has been employed as a steric constraint, even though it does not guarantee that clashes outside the recognition cavity do not happen. This is mainly true in cases of molecules with long side chains or relatively high molecular weights. Fulfillment of these criteria was best achieved by RBF. This vitamin appeared as a potential ligand 24 times during the VS studies and was a consensual ligand for PHP-01, -05, -08, and -10. Moreover, the RBF presented the highest fit value (4.90) among all other compounds.

RBF is a water-soluble vitamin from the B complex and is also known as vitamin B2. Chemically, it comprises a polyhydroxylated ribitol-like side chain and an isoalloxazine group, characteristic of flavones. RBF is found in eggs, milk, meat, and vegetables and appears yellow due to isoalloxazine ring resonance. Biologically, RBF is the main precursor for both flavin mononucleotide (FMN) and flavin adenine dinucleotide (FAD), key compounds in, for example, cellular respiration and redox reactions.^74^ RBF is provided through the diet but can also be biosynthesized by bacteria in the large intestine.^75,76^ For *Schistosoma*, RBF uptake may occur across the parasite’s tegument and/or via ingestion of the blood meal.^77^

**Figure 2:**
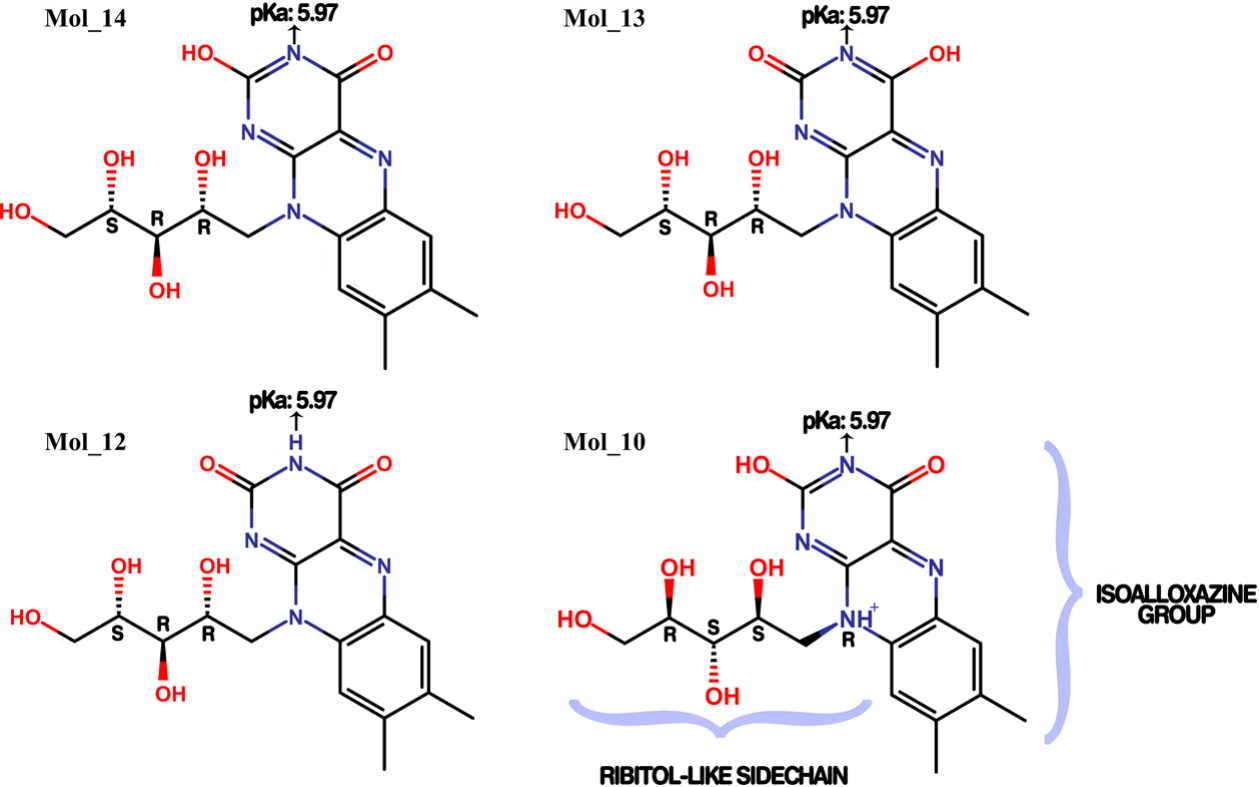
Riboflavin tautomers, isomers, and protomers pointed out as potential ligands of SmCB1. The p*K*_a_ values of these structures were assessed using Marvin Sketch 20.11 software. While the calculation resulted in a p*K*_a_ value of 5.97 for the N indicated by the arrow, experimental data shows a p*K*_a_ value of 10.2 for RBF (DrugBank ID: DB00140). Additionally, the analysis revealed that Mol_13 and Mol_14 are associated with physiological pH 7.4. Conversely, Mol_12 and Mol_10 are only present in strongly acidic environments (pH < 2).

RBF was identified as a potential ligand of SmCB1 even when assuming four structural variations in terms of tautomers, isomers, and protonation states, as illustrated in Figure 2. The result named Mol_14 was the most prevalent, and this had the highest FitValue. It is important to consider that all the different tautomers and protomers elected as potential ligands might be found within the host body, as the conditions for chemical species interconversions are fulfilled by blood pH, O_2_ levels, and temperature, as discussed in.^78^ Moreover, Mol_10 was the only protonated structure and also presented a different isomer of the ribitol-like group. This indicated that such isomerism is not crucial as well as the protonation state of the isoalloxazine ring (Figure 4, Mol 10).

Following on, as the pharmacophore models preserve the SmCB1 cavity coordinates, it is possible to visualize common interactions between the poses found for all four RBF structures (Supporting Information—Figure S4).

RBF interacted with some of the hotspots already described^28^ for the vinyl sulfones, including the residues of the catalytic triad Cys100, His270, and Asp290. However, RBF did not establish interactions with residues Leu146, Ala271, and HOH718, or with the water, HOH832, as was found for WRR-286. Separately, RBF potentially interacts with additional residues, including Ile193 and Leu252, as well as the water molecules HOH558 and HOH763.

In Supporting Information—Figure S4, the hydrogen bonds were analyzed as favorable or unfavorable considering the distances and angles. Most of the unfavorable hydrogen bonds were due to too short a distance between ligand and enzyme. This aspect can be solved by allowing the interacting molecules to achieve a more relaxed state.

Residues Gln94 and Gly269 (Supporting Information—Figure S5A) were especially important for hydrogen bonds in most of the screening data generated, suggesting that these are significant for recognizing RBF. An electrostatic interaction with residue Glu142 (Supporting Information—Figure S5B) was also prevalent and highlighted its importance to recognizing RBF as well as WRR-286. Finally, hydrophobic interactions involving several residues were also observed, among which Ile193 predominated (Supporting Information—Figure S5C).

### 3.4 Molecular Dynamics and Free Energy Calculations

To investigate the affinity of the RBF for SmCB1, a 1 μs MDS was performed. Piceid^79^ (PCD), a resveratrol derivative, which was identified during the VS as a poor SmCB1 binder, was used as a negative control. CRA555 (CRA), a vinyl sulfone analog,^28^ which did not inhibit SmCB1 at 100 μM,^28^ was also used as an experimental negative control. This compound was prepared as described in Section 2.3 “Datasets” for other ligands. CHARMM-GUI platform^48^ was used to prepare all three systems, including the free enzyme.

**Figure 3:**
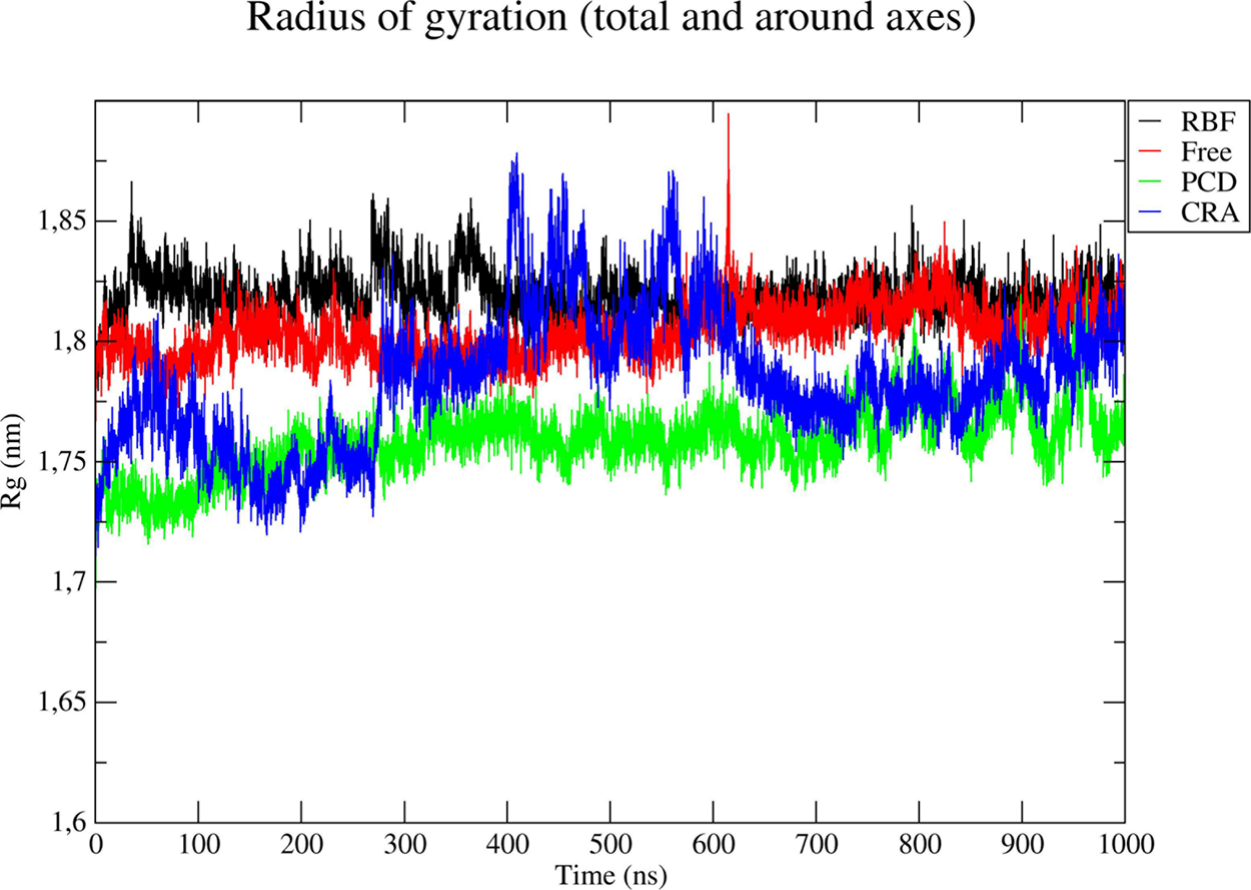
Radius of gyration for RBF (black), free enzyme (red), PCD (green), and CRA (blue).

The radius of gyration plot is shown in Figure 3 which is a measure of the overall structural integrity of the system during an MDS. As shown in red, the free enzyme remains relatively stable for about 600 ns, perhaps attributing to the highly flexible occluding loop. More details regarding this dynamic behavior are discussed below. The ligand-enzyme complex CRA555-SmCB1 has shown “dynamic instability” from 280 to 630 ns. Which aligns with this compound being a negative control.

**Figure 4:**
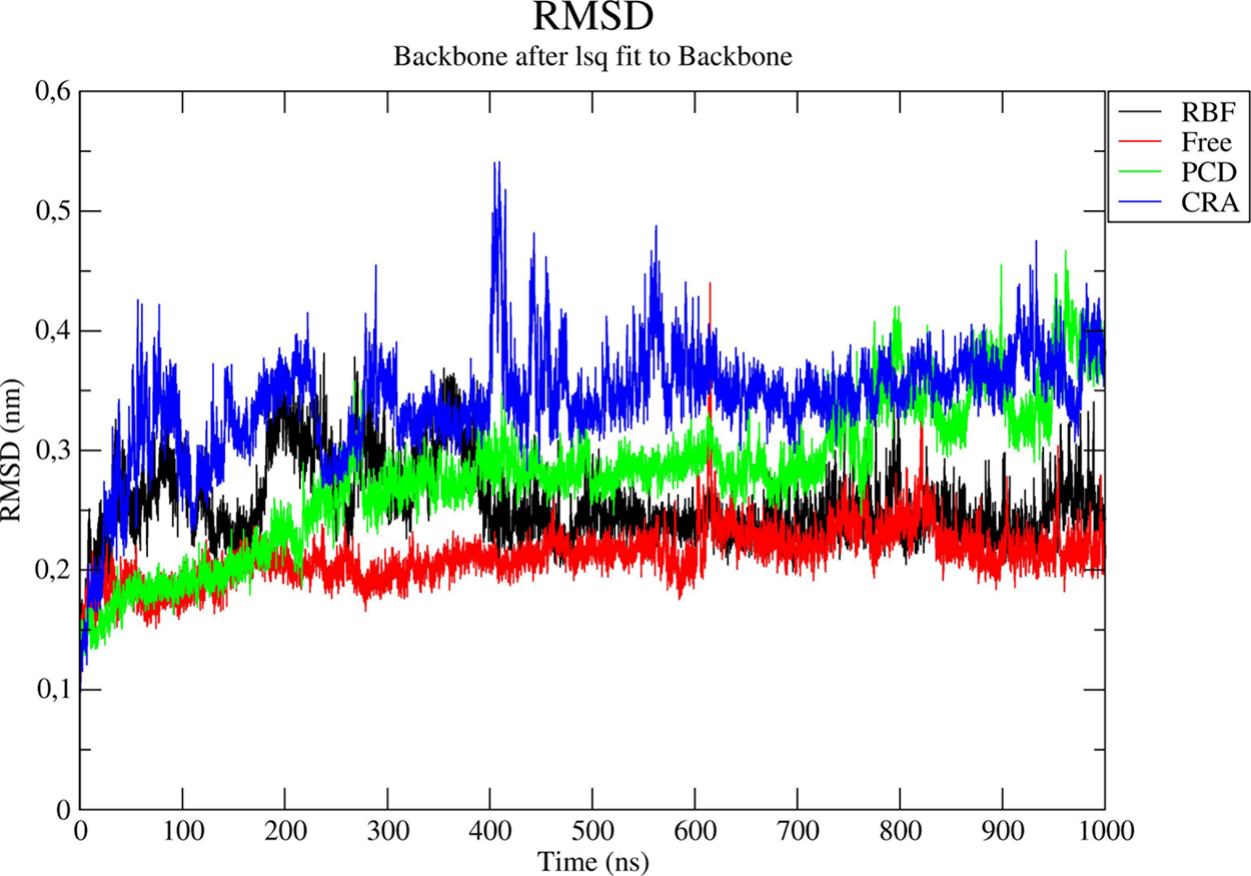
Root mean square deviation for RBF (black), free enzyme (red), PCD (green), and CRA (blue).

In MDS, root means square deviation (RMSD) is a measure of the overall structural stability as well as the dynamics profile of the main chain of a biomacromolecular system. As can be seen in Figure 4, RBF seems to induce structural changes in SmCB1 up to 400 ns. The enzyme then stabilizes (dynamic convergence). Regarding the PCD-SmCB1 complex, PCD was bound to SmCB1 up to about 50 ns. PCD was then released from the enzymatic active site (Supporting Information—Figure S6B). However, this ligand remains nearby the enzyme, and short-term clashes were observed between the ligand and enzyme (Supporting Information—Figure S6C). As shown in Figure 4, due to unfavorable ligand-enzyme interactions from 700 ns, the enzyme shows high structural instability. Finally, the CRA555-SmCB1 complex shows overall structural instability, and from about 270 ns, CRA555 is released from the active site and then immediately binds back into it. However, such a ligand-enzyme interaction is unfavorable. This profile is consistent with the data of Jilková et al. (2021), who measured no significant inhibition of SmCB1 activity by CRA555.^28^

**Figure 5:**
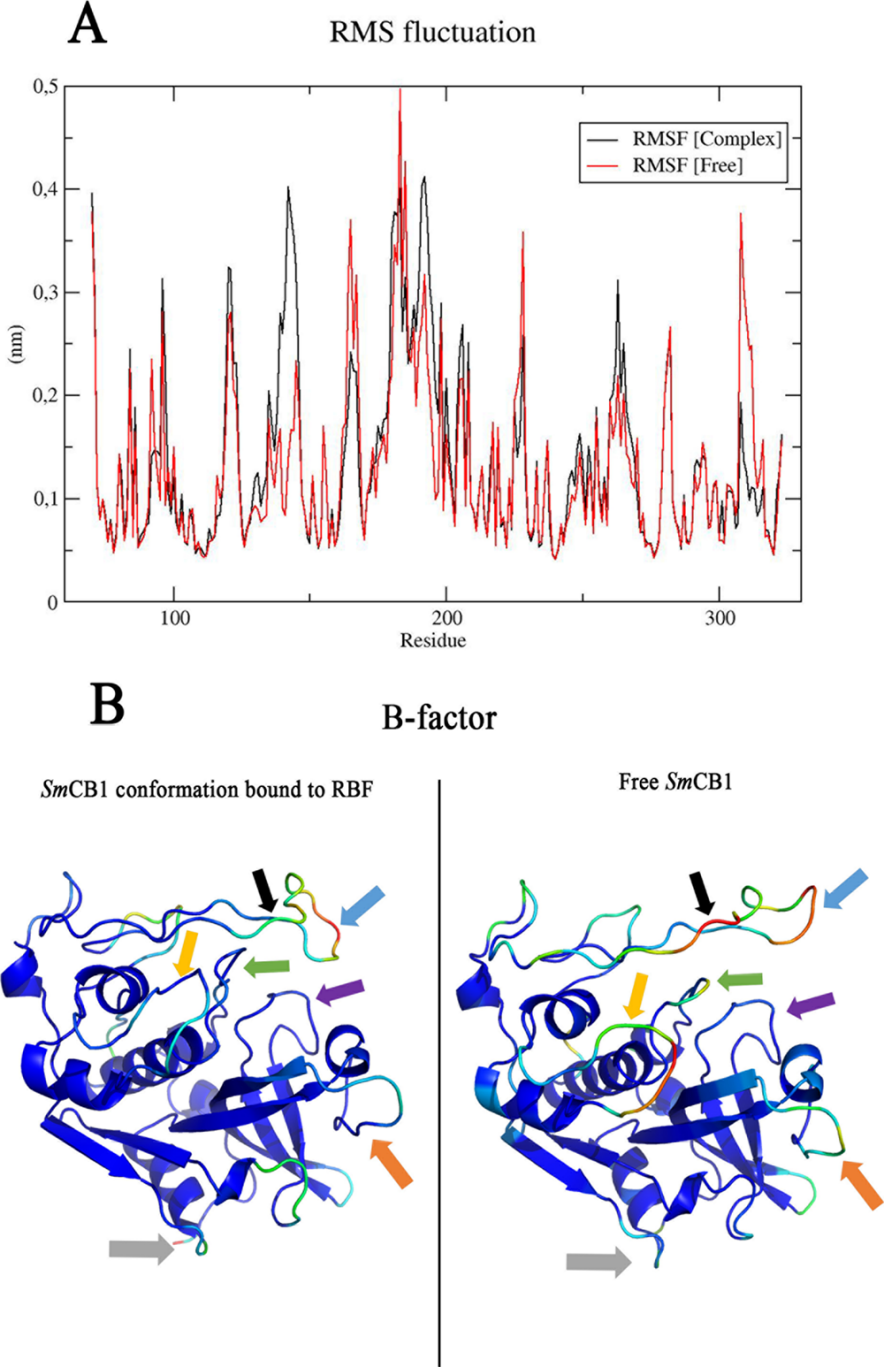
(A) RMSF chart constructed in XMGRACE. Black lines represent SmCB1 residue fluctuation along MDS bound to RBF; red lines represent free SmCB1. (B) Structures in ribbon with B-factor coloring (spectrum: range 4.67000 to 446.29999) were extracted via GROMACS and constructed for visualization in PyMol. Black and blue arrows: occluding loop. Green arrow, loop 1; yellow arrow, loop 2; orange arrow, loop 3; purple arrow, loop 4; and gray arrow, loop near the C-terminus (Arg 306 to Glu 316).

Figure 5 shows the RMSF (root mean square fluctuation) and B-factor (“temperature factor”) plots for SmCB1. RMSF is a measure of the overall structural flexibility of each residue of a protein. Additionally, the B-factor is a complementary way to investigate the dynamic profile of the system, whereby the protein structure is visualized in a color scheme, and more flexible (“hot”) residues are shown in red, and more rigid (“cold”) residues are shown in blue. From this point, just RBF, the relevant compound in this project, was considered.

The structure of the active site is composed of residues from four loops, including loops 1 and 2 belonging to the R domain and loops 3 and 4, which belong to the L domain.^80^ RBF causes a significant structural change in loop 2 (Glu135 to Pro148), mainly in residues Glu142, Gly143, and Gly144 (yellow arrow). In loop 1 (Thr90 to Cys100), residues Arg96 and Cys97 are the most affected by RBF in comparison to free protein (green arrow). Furthermore, the stretch from Gly190 to Tyr194, within the occluding loop (Phe175 to Tyr194), is more flexible when interacting with RBF compared with that in the free protein (black arrow). Also, in the occluding loop (blue arrow), it is important to highlight the greater flexibility of residues from His180 to Lys183, where His180 and His181 are known to be important for interacting with substrate.^81^ Greater flexibility is also observed in loop 3 (Lys260-Glu265, orange arrow). Less flexibility is observed between residues Glu310 and Ile313, which form a hairpin loop (gray arrow) when bound to RBF. Finally, loop 4 (Asn290 to Gly300) shows no significant conformational change upon interaction with RBF.

**Figure 6:**
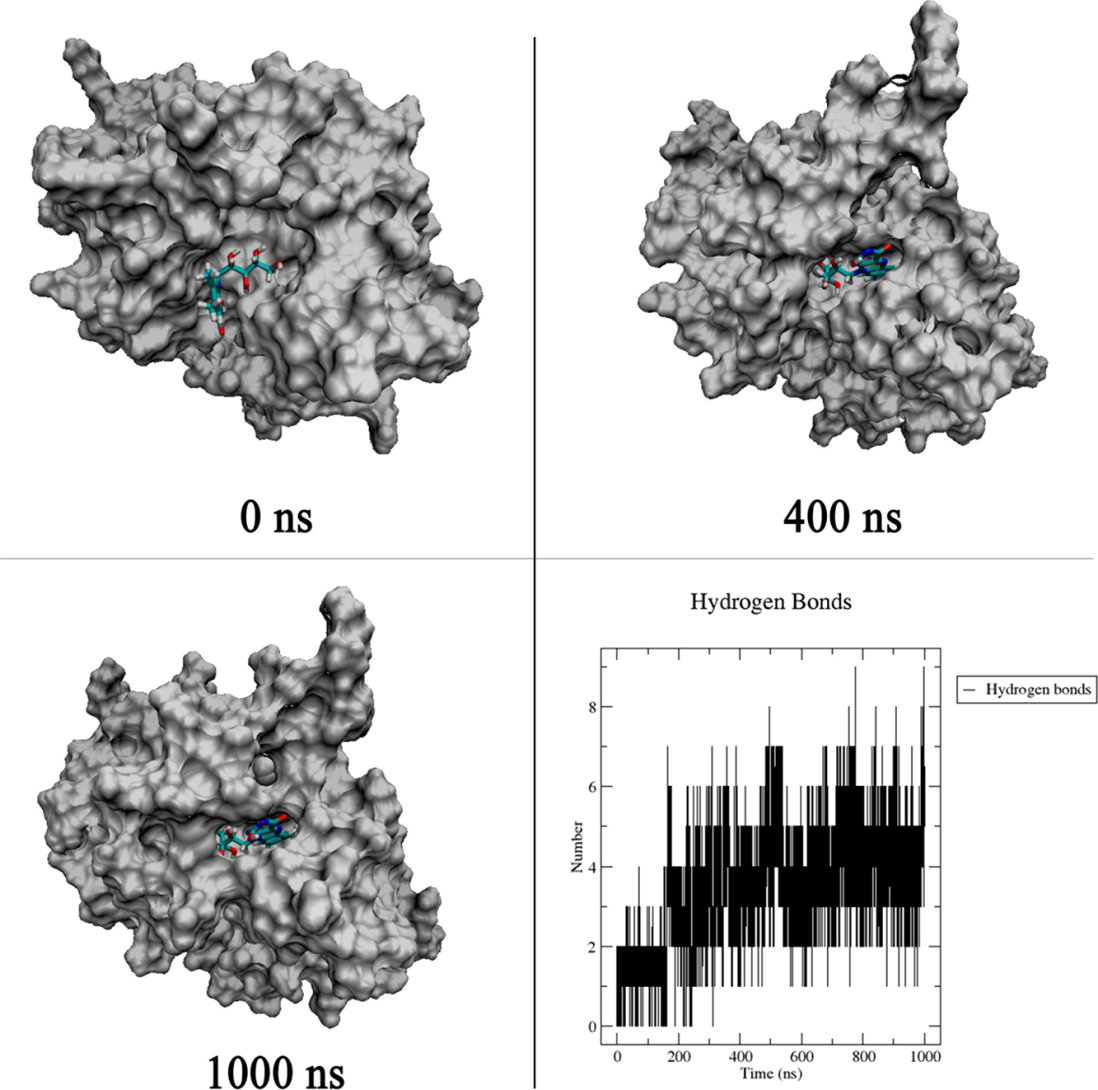
Snapshots of SmCB1 with RBF docked into the active site and the number of intermolecular hydrogen bonds as a function of the time of simulation.

Figure 6 shows the most relevant snapshots of the MDS. The solvent accessible surface of SmCB1 is shown gray, and the docked RBF is shown in the active site binding cavity. The chosen time simulations (0, 400, and 1000 ns) are based on the foregoing RMSD analysis in this section. As shown in Figure 4, the RBF-SmCB1 complex stabilizes at about 400 ns onward. At the beginning of the MDS run, the active cavity is open. After the stabilization of the complex at 400 ns, the cavity becomes narrow due to the engagement of a hydrogen-bond network (residue-RBF-residue), keeping the ligand enclosed in the cavity. Figure 6 shows the progressive increase in the number of hydrogen-bond interactions over time. During the simulation, the Hbond interactions formed between the RBF-SmCB1 complex remained within a range of 3–6 interactions most of the time, with occasional peaks of 8 or 9 interactions.

To quantify the intermolecular interaction and, consequently, the thermodynamic stability of the ligand-protein complex during MDS, it is good practice to calculate the corresponding free energy of binding. In this regard, the chosen time simulation window for the calculation was 400–1000 ns. As shown in Table 3, RBF shows a higher affinity to SmCB1 (a more negative value) than CRA, which corroborates the foregoing MDS structural analysis. Moreover, the enthalpic energy (Δ*H*) for CRA is positive, which is characteristic of unfavorable interactions,^82,83^ consistent with its use as a negative control during MDS.

**Table 3:** Free Energy of Binding for RBF and CRA555 with SmCB1^a^

energy components	RBF-SmCB1 (kcal/mol)	CRA555-SmCB1 (kcal/mol)
Δ*E* (VDW)	–48.24 ± 0.04	–35.54 ± 0.09
Δ*E* (EEL)	–49.5 ± 0.14	–10.65 ± 0.09
Δ*G* (GGAS)*	–97.74 ± 0.13	–46.19 ± 0.13
Δ*E* (EPB)	67.52 ± 0.13	27.75 ± 0.11
Δ*E* (ENPOLAR)	–32.24 ± 0.01	–25.65 ± 0.11
Δ*E* (EDISPER)	54.79 ± 0.002	48.28 ± 0.1
Δ*G* (GSOLV)**	90.07 ± 0.13	50.39 ± 0.13
Δ*H*	–7.67 ± 0.08	4.2 ± 0.05
–*T*Δ*S*	83.68 ± 1.09	84.77 ± 1.36
**Δ***G*_**binding**_	**–**91.35 ± 6.44	**–**80.57 ± 4.35

a Δ*E* (VDW): van der Waals energy; Δ*E* (EEL): electrostatic energy; Δ*E* (EPB): polar solvation energy; Δ*E* (ENPOLAR): repulsive contribution of the nonpolar solvation energy; Δ*E* (EDISPER): attractive contribution of the solvation energy. Δ*G* (GGAS) = Δ*E* (VDW) + Δ*E* (EEL) = total gas phase free energy. Δ*G* (GSOLV) = Δ*E* (EPB) + Δ*E* (ENPOLAR) + Δ*E* (EDISPER) = total solvation free energy. Δ*H*: enthalpy of binding; −*T*Δ*S*: conformational entropy after ligand binding. Δ*G*_binding_ = Δ*H* – *T*Δ*S* = free energy of binding.

### 3.5 RBF Inhibits SmCB1 and Human Cathepsin B and Kills Schistosomes In Vitro

**Figure 7:**
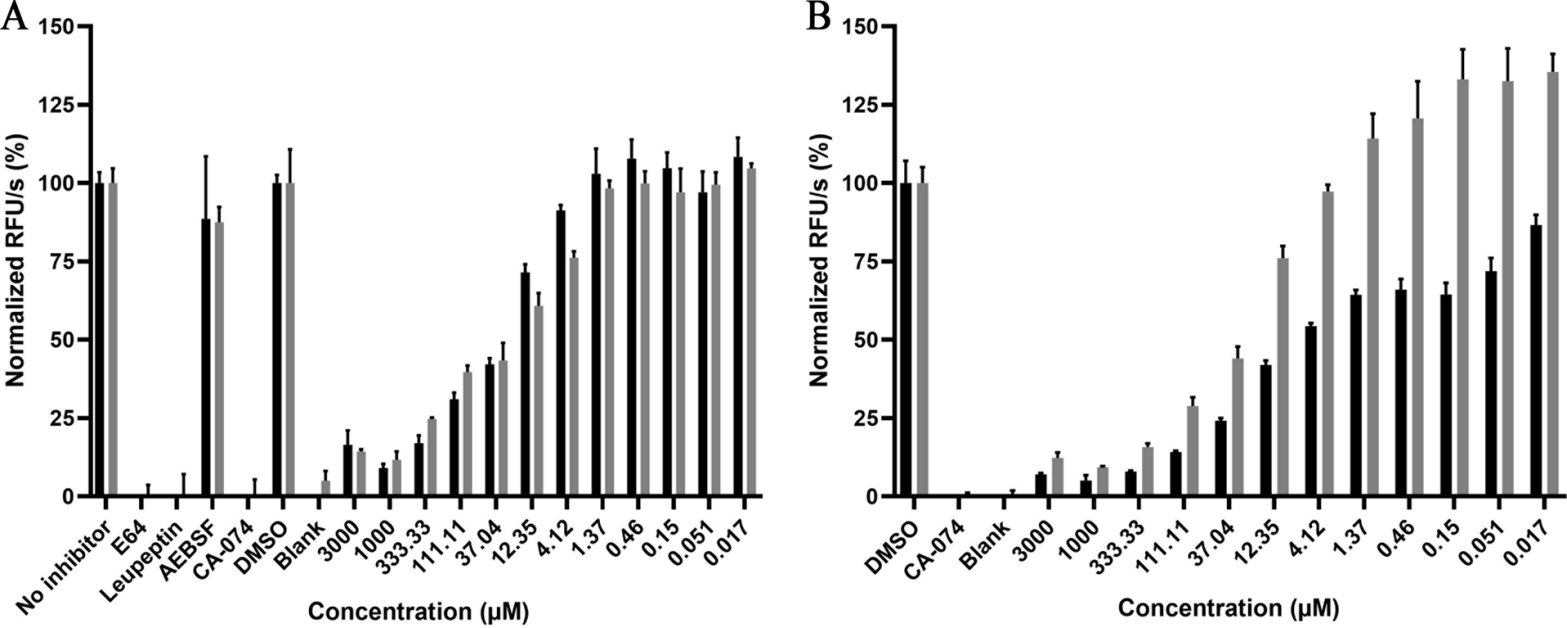
RBF inhibits SmCB1 and human cathepsin B in a concentration-dependent manner. Gray and black bars represent data in the presence and absence of 0.1% Triton X-100 for (A) SmCB1 and (B) human cathepsin B. The concentration of RBF, from 3000 to 0.017 μM, is indicated. Data, generated in the presence or absence of detergent, were normalized to the respective DMSO control: one biological assay performed in technical triplicate is shown.

The proposed RBF ligand was then experimentally tested for the inhibition of *SmCB1*. Specifically, acidic soluble extracts of adult *S. mansoni* were assayed in the presence of the reducing agent, DTT, and the dipeptidyl substrate, benzyloxy-carbonyl(Z)-RR-7-amido-4-methyl coumarin (AMC), conditions that essentially select for the major SmCB1 activity in the worm extract relative to other minor cathepsin activities, such as cathepsin L.^58,84^ Over 12 dilution points of riboflavin (3000–0.017 μM), the estimated IC_50_ value for inhibition was 20.50 ± 1.89 μM (Figure 7A). To account for possible aggregation of the ligand,^85−87^ we also performed the assay in the presence of 0.1% Triton X-100 detergent; however, the IC_50_ value was relatively unchanged (18.77 ± 2.13 μM). Importantly, to confirm that cathepsin B activity was being measured, we performed the assay after preincubation with the protease-class specific inhibitors E-64 (cysteine proteases), leupeptin (serine and cysteine proteases), AEBSF (serine proteases), and the cathepsin B-specific inhibitor, CA-074. All of these, except AEBSF, inhibited protease activity. Overall, therefore, the data suggest a *bone fide* concentration-dependent interaction between RBF and SmCB1. We extended these findings to human cathepsin B, which was also inhibited in a concentration-dependent manner in the absence and presence of detergent with similar IC_50_ values of 12.12 ± 1.86 and 13.11 ± 2.26 μM, respectively (Figure 7B). The data, therefore, suggest that the inhibition recorded here for RBF is a general phenomenon for cathepsin B.

**Figure 8:**
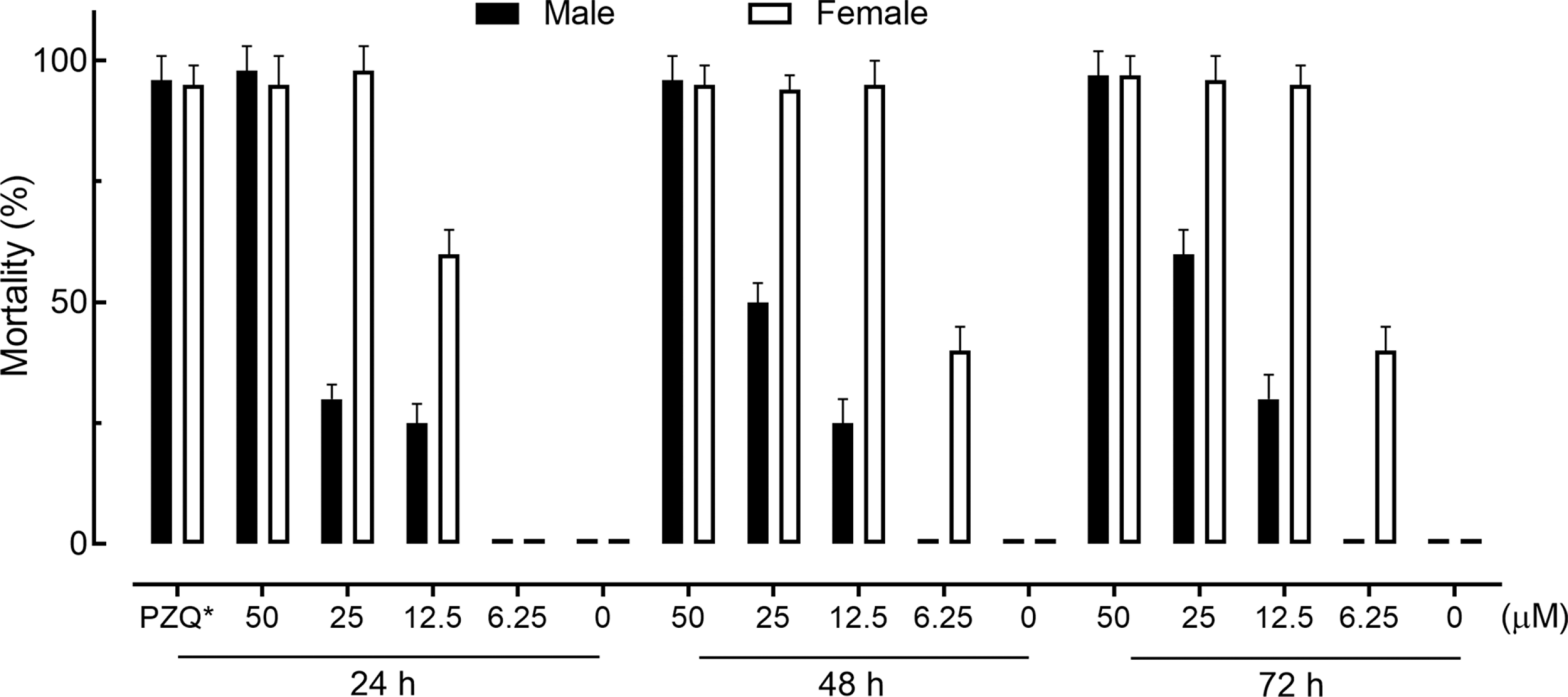
Mortality of *S. mansoni* in vitro maintained in the presence of different concentrations of RBF. Adult worms were perfused from mice 49 days after infection. Parasites were monitored for 24, 48, and 72 h. Values were normalized to DMSO controls (0 value at each time point) and represent means ± SD of three independent experiments each conducted in triplicate. (*) PZQ (praziquantel, the current drug for schistosomiasis treatment) at 2 μM.

Having confirmed inhibition of SmCB1 by RBF, the antischistosomal activity of RBF (50–6.25 μM), was assessed for up to 72 h against male and female adult *S. mansoni* maintained in vitro. As illustrated in Figure 8, female parasites were more susceptible to RBF compared to males. Specifically, 50 μM RBF (approximately 2.5 times the IC_50_ value for inhibition of SmCB1 (Figure 8)) killed all worms within 24 h. When the concentration was decreased to 25 or 12.5 μM, all female worms died within 24 h, whereas a significant portion of male worms remained viable for up to 72 h.

### 3.6 Oral Treatment of Mice Infected with *Schistosoma mansoni* Decreases Worm and Egg Burdens

Given the requirement for oral delivery of antischistosomal drugs,^27^ RBF was orally administered to mice harboring adult *S. mansoni* infections 42 days postinfection. A single dose of 400 or 100 mg/kg daily for seven consecutive days was administered. PZQ, at 400 mg/kg and solubilized in ethanol, 2% (v/v), served as the positive control. On day 63 postinfection, euthanasia was performed, and the number of worms and intestinal and fecal eggs was counted.

Treatment with one oral dose of 400 mg/kg RBF resulted in nonsignificant reductions in female and total worm burdens (13–16%; Table 4). However, the intestinal and fecal egg burdens were significantly decreased (20–27%, *P* < 0.05). In comparison, PZQ at 400 mg/kg achieved 90% reductions in both worm and egg burdens (*P* < 0.001). When RBF was administered daily for 7 days at 100 mg/kg, significant, if modest, reductions in total (23.5%, *P* < 0.05) and female (26.4%, *P* < 0.05) worm burden were recorded. However, this was accompanied by a substantial decrease in the number of eggs in the intestine (91.4%, *P* < 0.001) and feces (73.6%, *P* < 0.001), a result that was reproduced in a second experiment.

**Table 4:** Efficacy of RBF and PZQ in a Mouse Model of *S. mansoni* Infection^a^

	dose (mg/kg)	female worm burden reduction (%)	total worm burden reduction (%)	intestinal egg burden reduction (%)^d^	fecal egg burden reduction (%)
RBF	400^b^	16.2 (±8.8)	13.6 (±6.3)	27.1 (±8.4) *	19.8 (±5.4) *
	7 × 100^b^	26.4 (±5.2) *	23.5 (±4.5) *	91.4 (±7.2) ***	73.6 (±4.8) ***
	7 × 100^c^	21.9 (±6.3) *	19.1 (±5.7) *	80.3 (±5.9) ***	80.1 (±7.3) ***
PZQ	400^b^	90.7 (±8.1) ***	91.5 (±7.8) ***	88.9 (±6.7) ***	91.3 (±7.5) ***

a Values represent means ± SD values (*n* = 5) and were normalized to mouse groups treated with vehicle alone. *P < 0.05 and ***P < 0.001.

b Experiment 1.

c Experiment 2.

d Immature eggs in the intestine were assessed by oogram analysis.

Thus, the data from the mouse model of *S. mansoni* infection indicate that the single high-dose approach is less effective than the longer regimen involving lower daily dosing, which may suggest that the vitamin has an accumulative effect on the parasite’s survival and egg production capacity. Under the longer dosing regimen, RBF is orally effective by modestly decreasing worm burdens but greatly impeding (>80%) egg production. This encourages increasing and/or prolonging the dosing regimen to further decrease worm and egg burdens and thereby decrease morbidity. Similar experiments should also be considered in animal infection models of *Schistosoma japonicum* and *Schistosoma hematobium*, the two other medically important schistosomes.

### 3.7 Critical Analysis

Based on the evidence shown here for the activity of riboflavin in a mouse model of *S. mansoni* infection, it may be possible to consider a repurposing opportunity involving clinical trials with *Schistosomiasis mansoni* patients, whereby the vitamin is taken under various dosing regimens with monitoring of fecal egg burdens to measure drug efficacy. On the plus side, RBF is a ubiquitous health supplement, cheap and easy to make via microbial production,^88^ and essentially nontoxic: the LD_50_ (50% lethal dose) values in rats are 0.6, 5, and >10 g/kg when administered by the intraperitoneal, subcutaneous, and oral routes, respectively.^89^ Also, RBF has anti-inflammatory and antioxidant properties.^90^ However, a key consideration is that absorption of RBF across the human intestine involves active transport which is saturable,^91,92^ and would limit the blood levels of RBF achievable. For example, studies have shown in human volunteers that the maximum blood concentration of riboflavin achievable is 200–300 nM after a 20, 40, or 60 mg oral dose,^91^ which is ∼80-fold less than the IC_50_ values measured here for inhibition of SmCB1 by the vitamin. Assuming that the uptake of RBF by the mouse gut is likewise saturable with nanomolar levels of circulating RBF (we could not find data for the PK attributes of RBF in mice), it may well be that the antischistosomal activity measured in our mouse model is, in whole or part, disconnected from the inhibition of SmCB1 and that other, as yet unknown, mechanisms are involved. It is also possible that the antischistosomal effect of RBF is due to its accumulation by the worm, as preliminarily suggested here, in which case it may prove useful to take multiple daily doses of RBF in the hope that this translates into measurable reductions in fecal egg outputs and an associated decrease in disease-related morbidity.

## 4 Conclusions

Using a pharmacophore-based VS approach with an FDA-approved drug data set, we discovered RBF as a potential inhibitor of SmCB1, the major proteolytic enzyme and well-studied drug target, in the gut of the schistosome parasite. RBF established eight hydrogen-bond interactions with the SmCB1 along the 1 μs MDS, during which RBF was trapped within the protease’s binding cavity; otherwise, the structural integrity of the protease was unaltered. The prediction of RBF as a ligand for SmCB1 was confirmed in a biochemical inhibition assay and extended to include human cathepsin B. Further, RBF was schistosomicidal in vitro and, in a murine model of *S. mansoni* infection, significantly decreased worm and, egg burdens. Our report delineates a path from the in silico prediction of a target-ligand interaction to a demonstration of in vivo efficacy in an animal infection model for a globally important disease of poverty that has been reliant on just one drug therapy for over 40 years.

## Supporting Information Available

Additional experimental details and methods; ROC curve equation and AUC depiction; retrieved riboflavin conformations from virtual screening; pharmacophore model; processing of datasets for pharmacophore validation and VS; selected pharmacophore models; derivation scheme of vinylsulfonic ligands; analysis of energy components of the RBF-SmCB1 interactions; interactions of the identified conformations of riboflavin; and piceid location during dynamics (PDF)
