# Supplementary material for: Pharmacophore Virtual Screening Identifies Riboflavin as an Inhibitor of the Schistosome Cathepsin B1 Protease with Antiparasitic Activity
Source: ACS Omega. 2024 May 30;9(23):25356–69. doi: 10.1021/acsomega.4c03376 (PMC11170711; doi:10.1021/acsomega.4c03376)
Supplement: Supplementary file 1 — ao4c03376_si_001.pdf [file ao4c03376_si_001.pdf]

Pharmacophore virtual screening identifies riboflavin as an inhibitor of the schistosome cathepsin B1 protease with antiparasitic activity

Ramon M. Cogo[a], Thaís F. A. Pavani[a], Ana C. A. Mengarda[b], Rayssa A. Cajas[b], Thainá R. Teixeira[b], Lucas Fukui-Silva[b], Yujie Uli Sun[c], Lawrence J. Liu[c], Dilini K. Amarasinghe[c], Michael C. Yoon[c], Osvaldo A. Santos-Filho[d], Josué de Moraes[b1], Conor R. Caffrey[c], Daniela G. G. Rando[e*]

[a]Universidade Federal de São Paulo - Campus Diadema, Curso de Pós-Graduação em Biologia Química da Unifesp, Rua São Nicolau 210, 2º andar, Centro, Diadema, São Paulo, BR

[b]Universidade Guarulhos, Núcleo de Pesquisa em Doenças Negligenciadas – NPDN, Praça Tereza Cristina 88, Guarulhos, BR.

[c]University of California San Diego, Center for Discovery and Innovation in Parasitic Diseases, Skaggs School of Pharmacy and Pharmaceutical Sciences, La Jolla, CA, US.

[d]Universidade Federal do Rio de Janeiro, Instituto de Pesquisas de Produtos Naturais Walter Mors, Av. Carlos Chagas Filho, 373, Bloco H, Rio de Janeiro, BR[2].

[e]Universidade Federal de São Paulo - Campus Diadema, Grupo de Pesquisas Químico-Farmacêuticas da Unifesp, Department of Pharmaceutical Sciences Rua São Nicolau, 210, 2º andar, Centro, Diadema, São Paulo, BR.

*****Corresponding author

E-mail address: dgrando@unifesp.br (D.G.G, Rando)

[1] Núcleo de Pesquisa em Negligenciadas, Instituto Científico e Tecnológico, Universidade Brazil - São Paulo, Brazil.

SUPPLEMENTARY MATERIAL

Table S1. ROC curve equation and AUC depiction.

**Class**	*Positive*	*Negative*
Positive instance	True Positive (TP)	False Positives (FP)
Negative Instance	False Negatives (FN)	True Negatives (TN)
$TP\ rate = \frac{TP}{P}$ — True Positive rate.	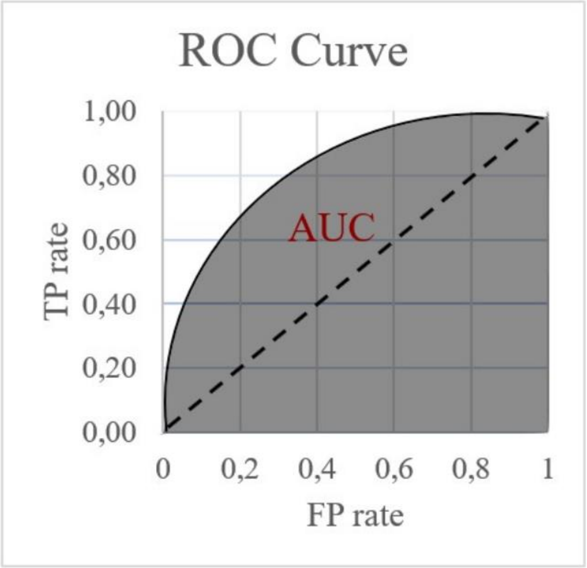	
$FP\ rate = \frac{FP}{P}$ — False Positives Rate.		

Table S2. Retrieved riboflavin conformations from virtual screening.

PHP-01

01_Mol-14

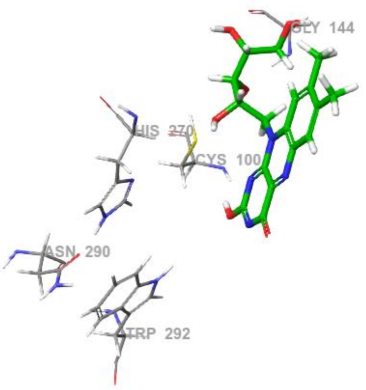

PHP-05

01_Mol-14	02_Mol-14	03_Mol-10
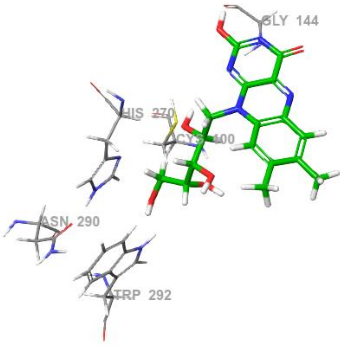	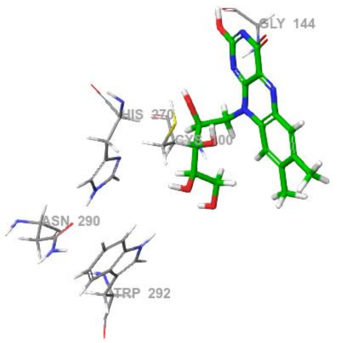	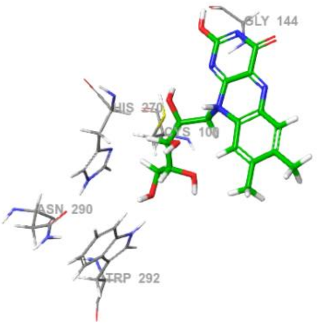

04_Mol-14	05_Mol-10	06_Mol-14
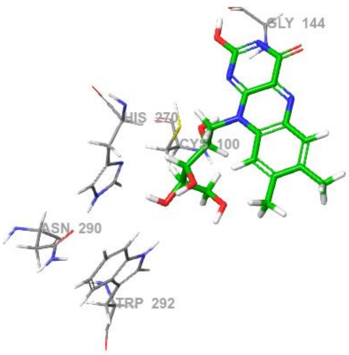	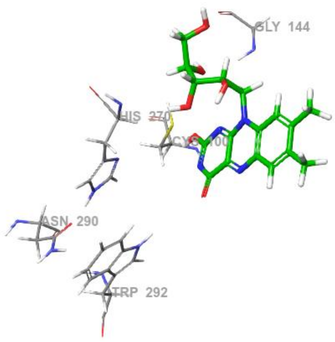	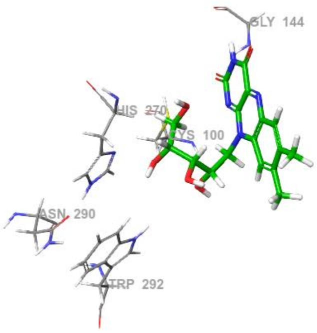

Table S2. Retrieved riboflavin conformations from virtual screening (continuing).

07_Mol-14	08_Mol-10	09_Mol-14
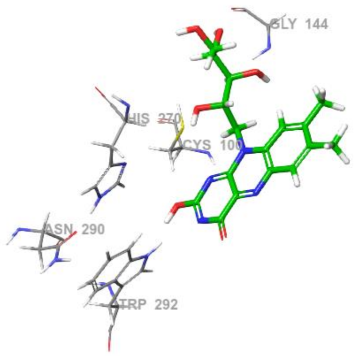	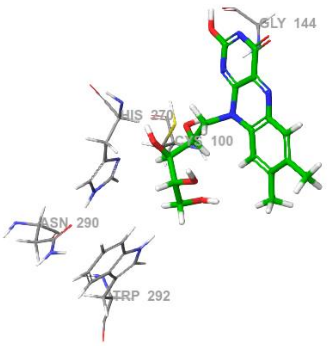	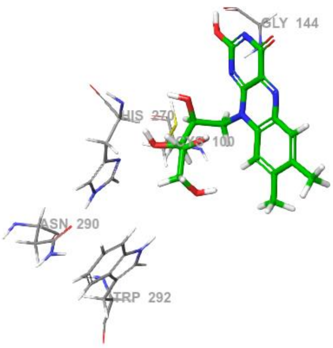

PHP-08

01_Mol-14	02_Mol-14	03_Mol-13
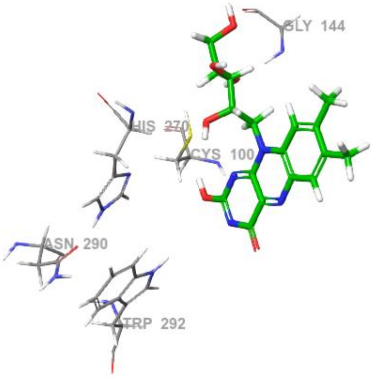	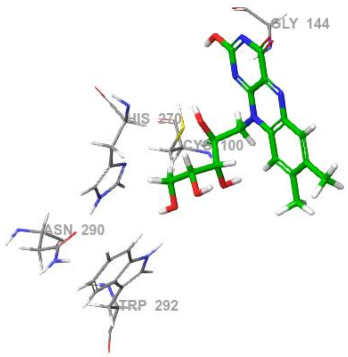	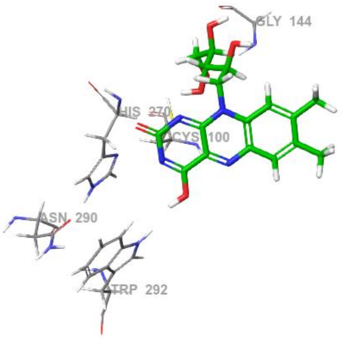
04_Mol-10	05_Mol-14	06_Mol-13
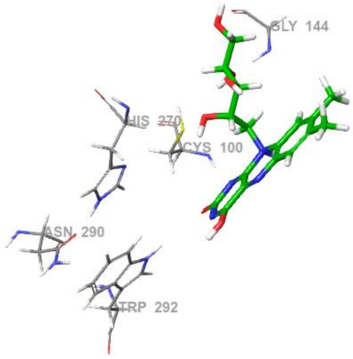	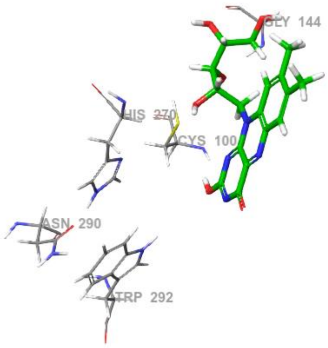	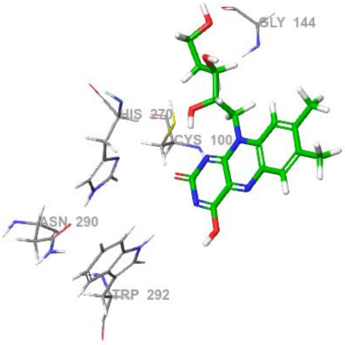

Table S2. Retrieved riboflavin conformations from virtual screening (continuing).

07_Mol-12	08_Mol-13	09_Mol-14
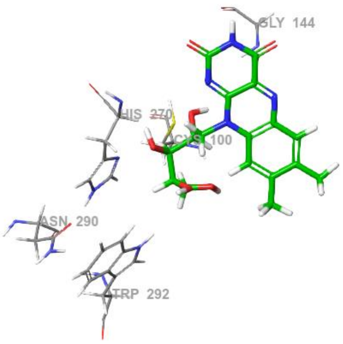	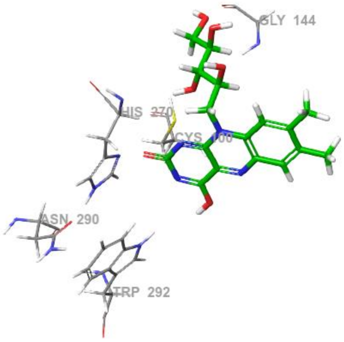	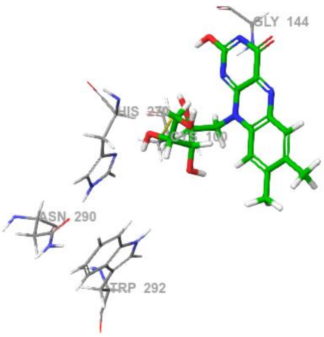
10_Mol-14		
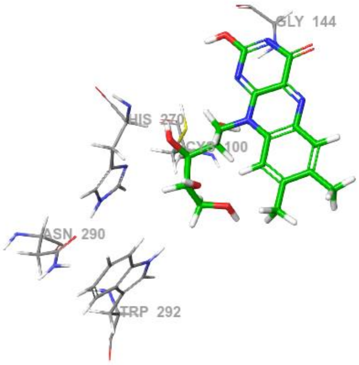		

PHP-10

01_Mol-13	02_Mol-13	03_Mol-10
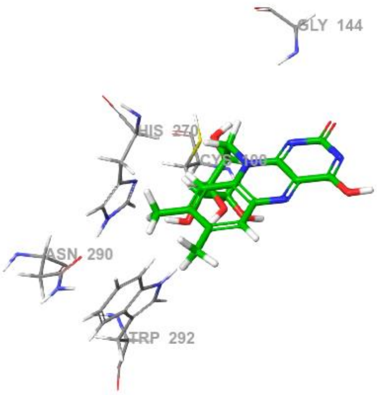	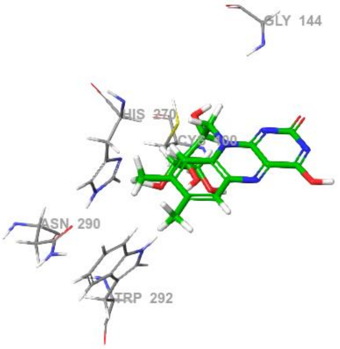	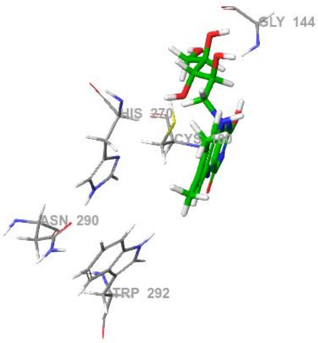

Table S3. Pharmacophore model.

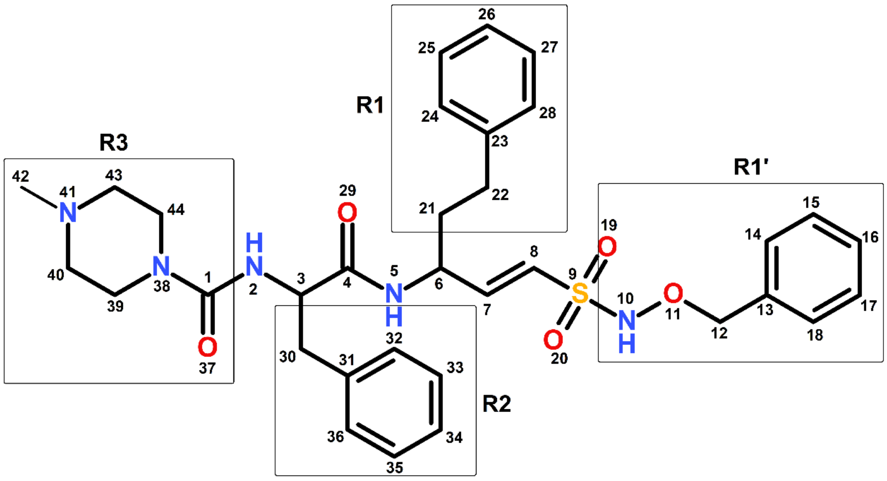

Model	Features						
PH-01	HBDon-02	HBDon-05	HBAc-29	HBAc-19	HBAc-20	HBDon-10	-
PH-02	HBDon-02	HBDon-05	HBAc-29	HBAc-19	HBAc-20	HBDon-10	-
PH-03	HBDon-02	HBDon-05	-	HBAc-19	HBAc-20	HBDon-10	Hyd-R2
PH-04	HBDon-02	HBDon-05	-	HBAc-19	HBAc-20	HBDon-10	Hyd-R1’
PH-05	HBDon-02	HBDon-05	-	HBAc-19	HBAc-20	HBDon-10	Hyd-R1
PH-06	HBDon-02	HBDon-05	HBAc-29	HBAc-19	-	HBDon-10	Hyd-R2
PH-07	HBDon-02	HBDon-05	HBAc-29	HBAc-19	-	HBDon-10	Hyd-R1’
PH-08	HBDon-02	HBDon-05	HBAc-29	HBAc-19	-	HBDon-10	Hyd-R1
PH-09	HBDon-02	HBDon-05	-	HBAc-19	HBAc-20	HBDon-10	Hyd-R2
PH-10	HBDon-02	HBDon-05	-	HBAc-19	HBAc-20	HBDon-10	Hyd-R1’

HBDon and HBAc are hydrogen bond donors and acceptors, respectively. The numbering is related to the chemical group position from the ligand that establishes the contact. The aromatic/hydrophobic features (Hyd) were named according to the aromatic groups linked to the main vinyl sulfone chain, as illustrated in the scaffold above, and were named as Hyd-R2, Hyd-R1 and Hyd-R1’. Pharmacophore images are displayed in the Supplementary Material Figure 1.

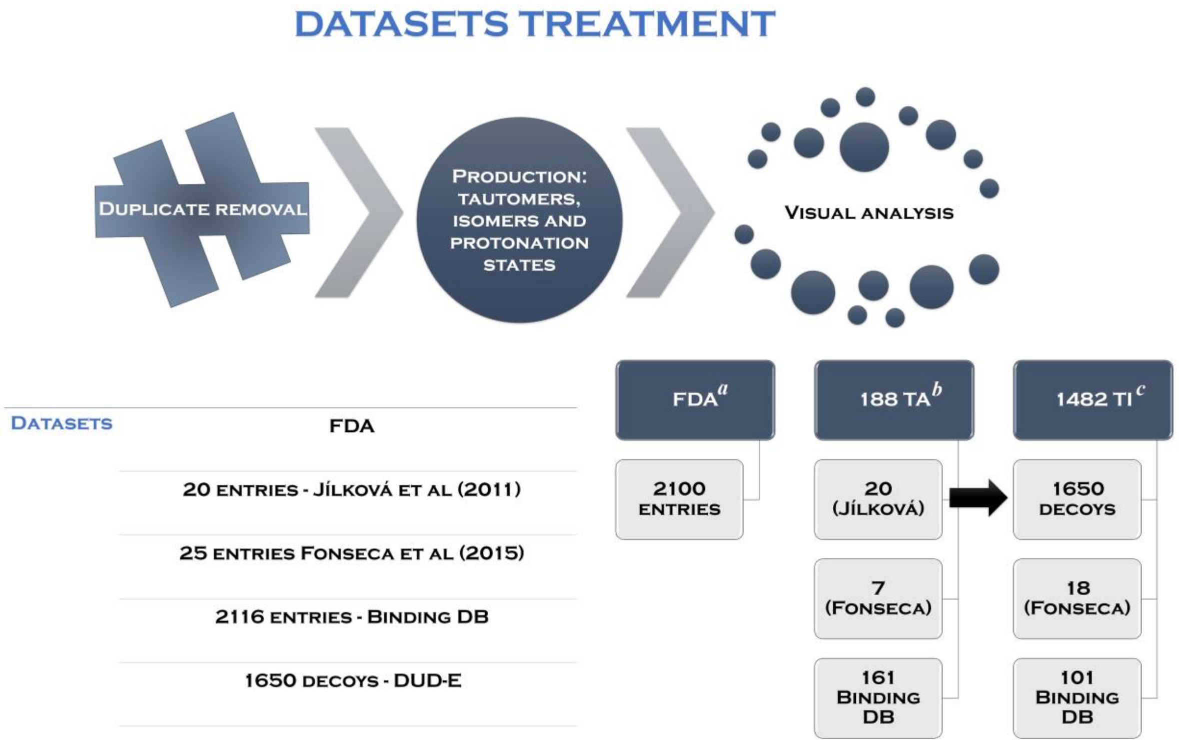

Figure S1. Processing of datasets for pharmacophore validation and VS. The black arrow indicates that the 20 analogues that were used as references for modeling the decoys. a) FDA, Food and Drug Administration; b) TA, Total Actives, and c) TI, Total Inactives.

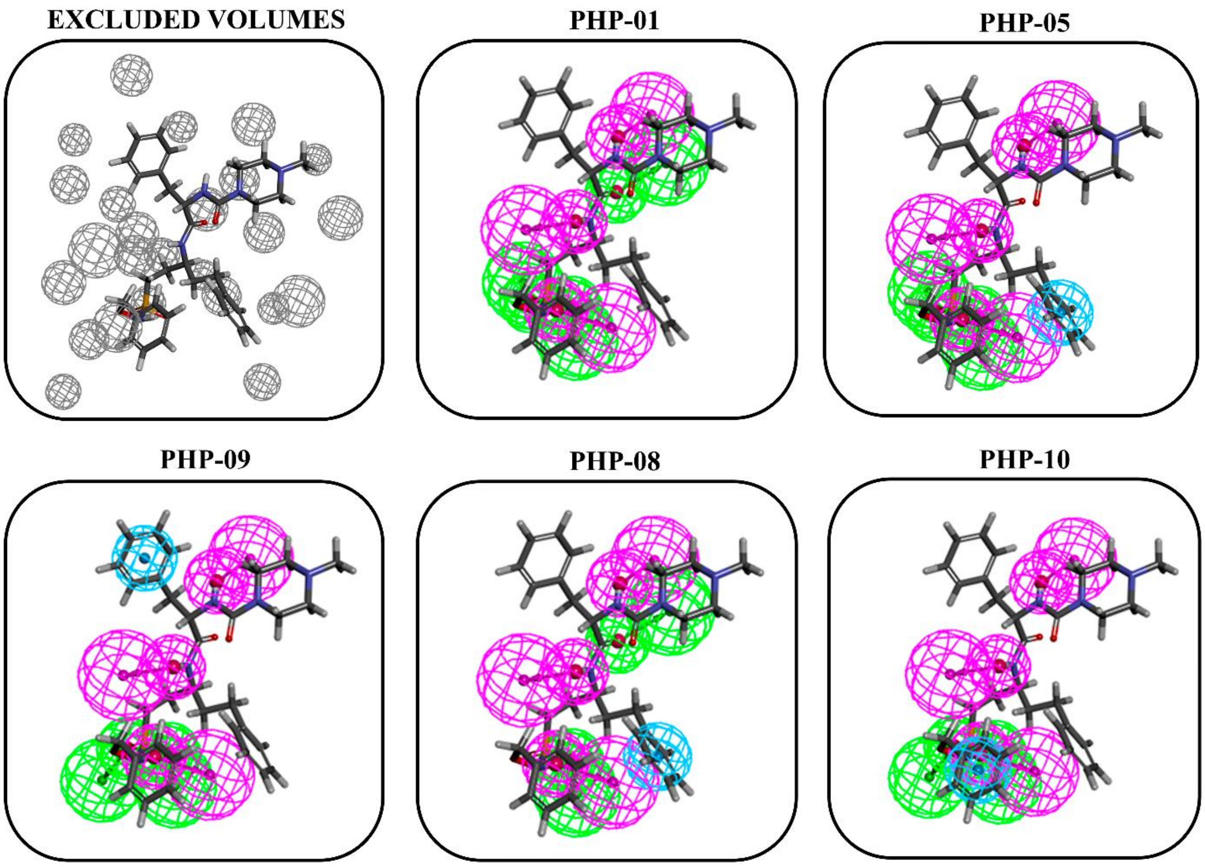

Figure S2. Selected pharmacophore models. Excluded volumes feature are represented in grey. Hydrogen bond acceptor features are in magenta. Hydrogen bond donor features are in green. Hydrophonic/Aromatic features are in cyan. Smaller spheres in the models are the interaction regions considering the ligand, while the greater spheres are the interaction regions considering residues in the *Sm*CB1 cavity.

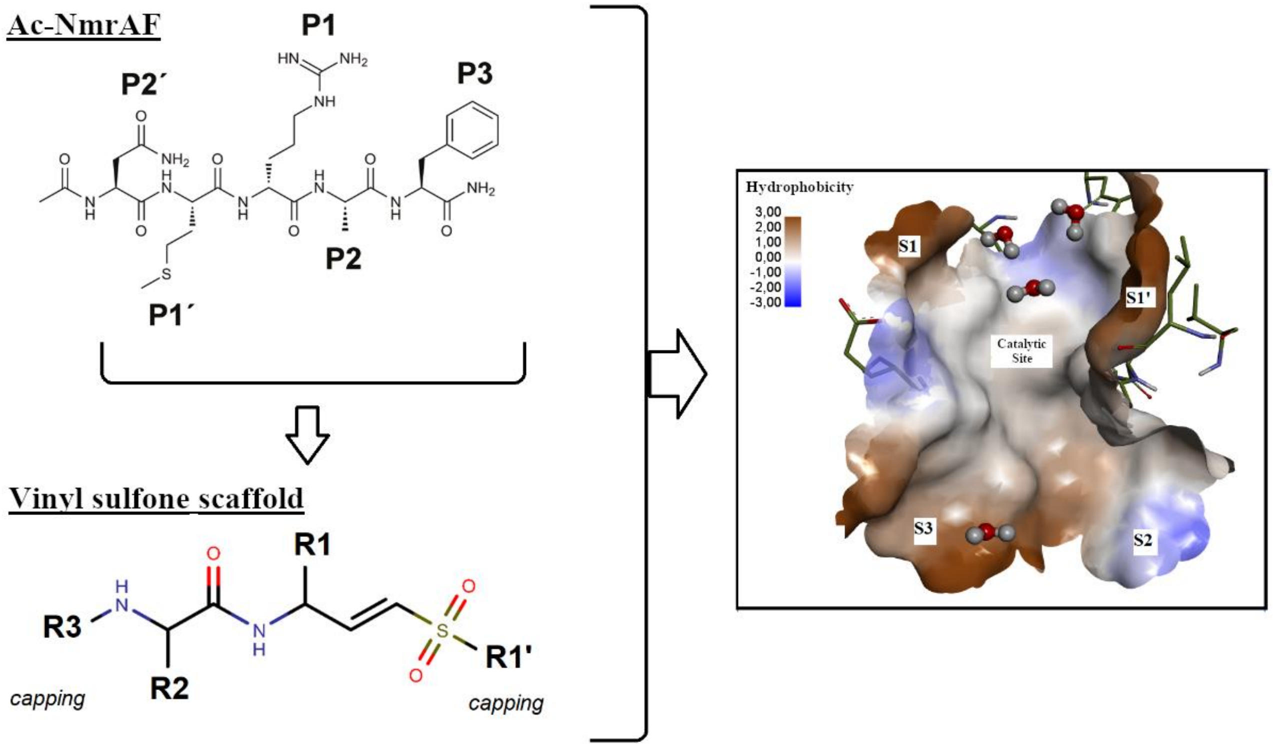

Figure S3. Derivation Scheme of Vinylsulfonic Ligands. Based on *Sm*CB1 inhibitor peptides, Jilkova et al. derived a series of vinylsulfonic ligands with substituents R1', R1, R2, and R3 designed to potentially interact with the enzyme's S1', S1, S2, and S3 sites, respectively.

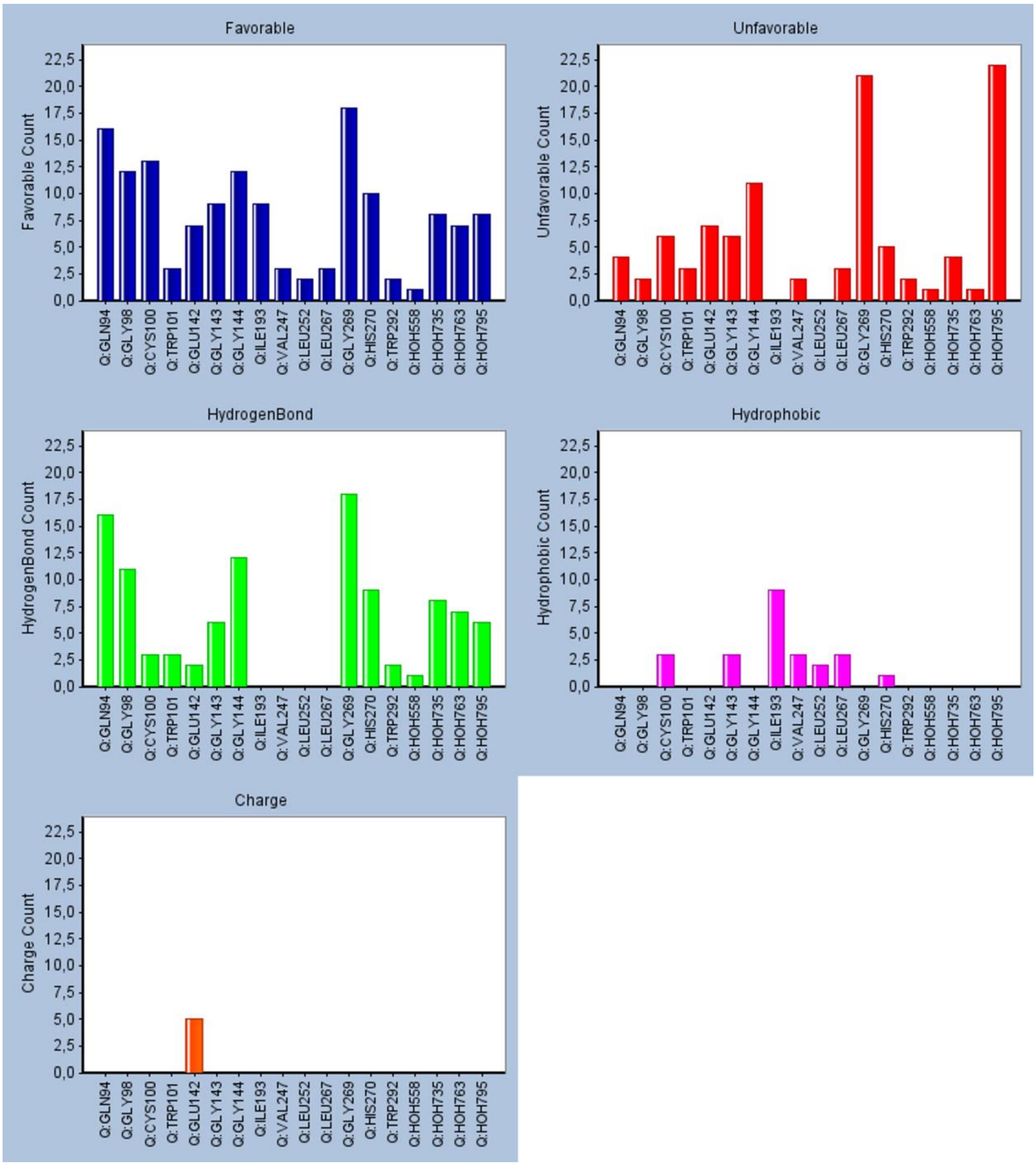

Figure S4. Analysis of energy components of the RBF-SmCB1 interactions. Bar hights is a function of the number ("frequency") of poses sampled from the VS procedure.

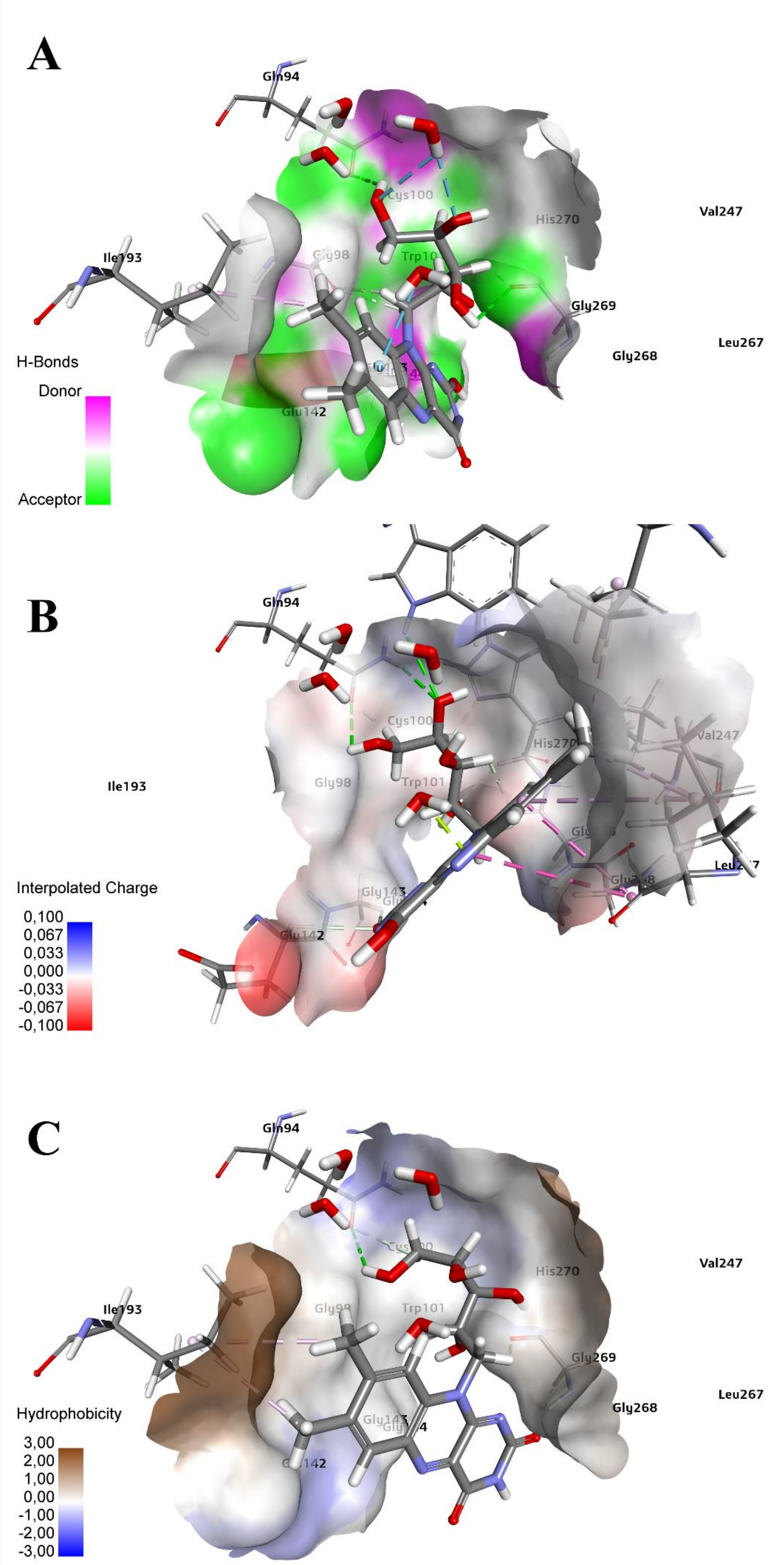

Figure S5 – Interactions of the identified conformations of riboflavin. **A.** 01_Mol-14 (PHP-05, supplementary material Figure 1) interactions based on the hydrogen bond donor and acceptor surface. **B.** 01_Mol-13 (PHP-10, supplementary material Figure 1) interactions regarding the charge surface. **C.** 07_Mol-12 (PHP-08, supplementary material Figure 1) interactions regarding the hydrophobic surface.

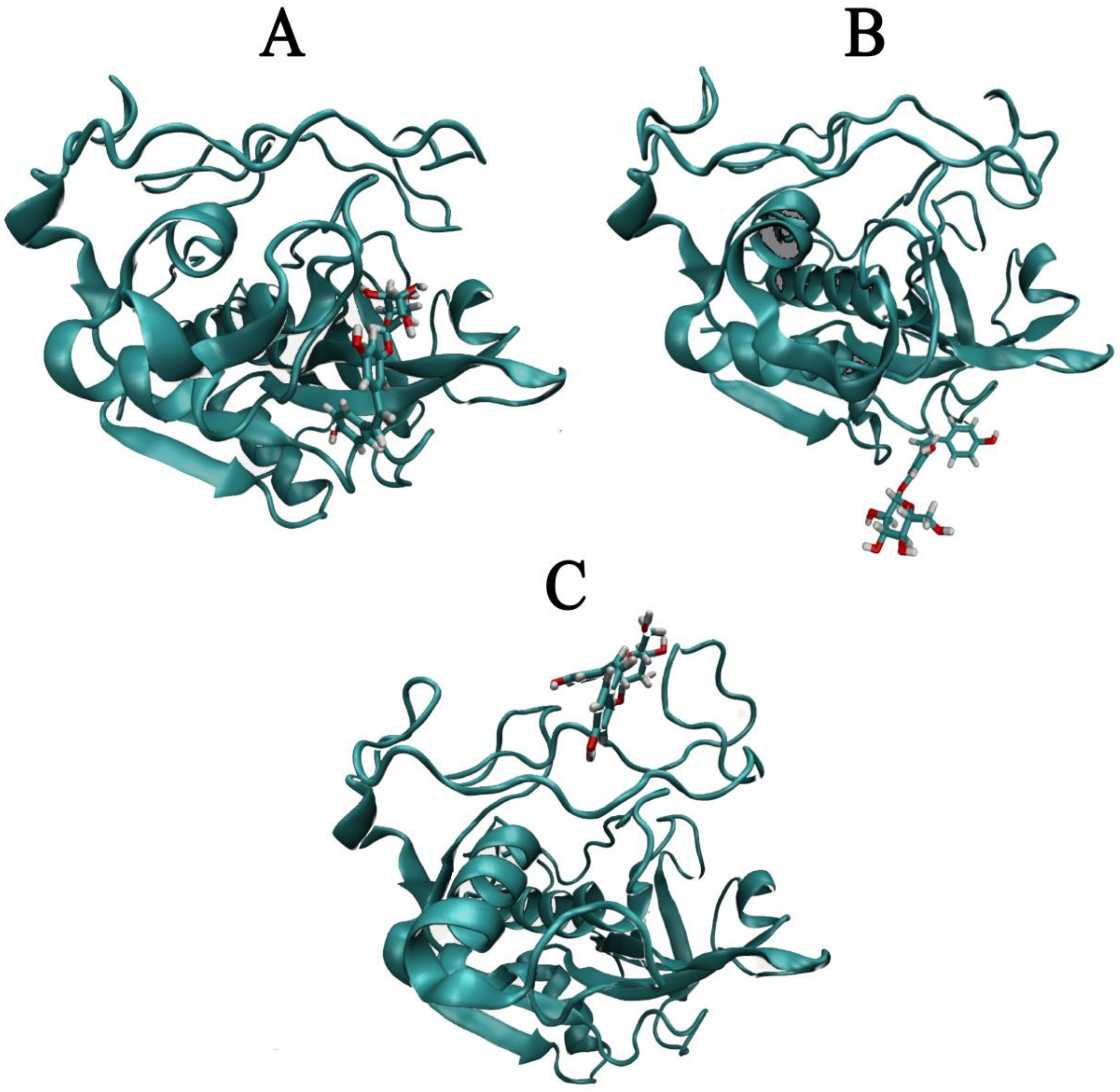

Figure S6. Piceid location during dynamics. A. Starting point, at 0 ns. **B.** Nearly 50 ns, when interaction is lost. **C.** Loop contact.
